# Pharmaceutical targeting of the cannabinoid type 1 receptor impacts the crosstalk between immune cells and islets to reduce insulitis in humans

**DOI:** 10.1007/s00125-024-06193-6

**Published:** 2024-06-12

**Authors:** Elise Wreven, María Soledad Ruiz de Adana, Stéphan Hardivillé, Valery Gmyr, Julie Kerr-Conte, Mikael Chetboun, Gianni Pasquetti, Nathalie Delalleau, Julien Thévenet, Anaïs Coddeville, María José Vallejo Herrera, Liad Hinden, Inmaculada Concepción Benavides Espínola, Mireia Gómez Duro, Lourdes Sanchez Salido, Francisca Linares, Francisco-Javier Bermúdez-Silva, Joseph Tam, Caroline Bonner, Josephine M. Egan, Gabriel Olveira, Natalia Colomo, François Pattou, Isabel González-Mariscal

**Affiliations:** 1 Inserm UMR1190 – Translational Research for Diabetes, Université de Lille, CHU Lille, Institut Pasteur de Lille, Inserm, European Genomic Institute for Diabetes, Lille, France; 2 Servicio de Endocrinología y Nutrición, Hospital Regional Universitario de Málaga, Instituto de Investigación Biomédica de Málaga y Plataforma en Nanomedicina-IBIMA-Plataforma BIONAND, Málaga, Spain; 3 Centro de Investigación Biomédica en Red de Diabetes y Enfermedades Metabólicas Asociadas (CIBERDEM), Instituto de Salud Carlos III, Málaga, Spain; 4 CNRS UMR8576 – UGSF – Unité de Glycobiologie Structurale et Fonctionnelle, Université de Lille, Lille, France; 5 Obesity and Metabolism Laboratory, Institute for Drug Research, School of Pharmacy, Faculty of Medicine, The Hebrew University of Jerusalem, Jerusalem, Israel; 6 Laboratory of Clinical Investigation, National Institute on Aging, National Institutes of Health, Baltimore, MD USA; 7 Departamento de Medicina y Cirugía, Facultad de Medicina, Universidad de Málaga, Málaga, Spain; 8 Grupo de Trabajo de Investigación Básica en Diabetes, Sociedad Española de Diabetes, Madrid, Spain

**Keywords:** Cannabinoid receptor, CB1R, Endocannabinoid, Insulitis, Islet of Langerhans, Peripheral CB1R inverse agonist, Type 1 diabetes

## Abstract

**Aims/hypothesis:**

Insulitis, a hallmark of inflammation preceding autoimmune type 1 diabetes, leads to the eventual loss of functional beta cells. However, functional beta cells can persist even in the face of continuous insulitis. Despite advances in immunosuppressive treatments, maintaining functional beta cells to prevent insulitis progression and hyperglycaemia remains a challenge. The cannabinoid type 1 receptor (CB1R), present in immune cells and beta cells, regulates inflammation and beta cell function. Here, we pioneer an ex vivo model mirroring human insulitis to investigate the role of CB1R in this process.

**Methods:**

CD4^+^ T lymphocytes were isolated from peripheral blood mononuclear cells (PBMCs) from male and female individuals at the onset of type 1 diabetes and from non-diabetic individuals, RNA was extracted and mRNA expression was analysed by real-time PCR. Single beta cell expression from donors with type 1 diabetes was obtained from data mining. Patient-derived human islets from male and female cadaveric donors were 3D-cultured in solubilised extracellular matrix gel in co-culture with the same donor PBMCs, and incubated with cytokines (IL-1β, TNF-α, IFN-γ) for 24–48 h in the presence of vehicle or increasing concentrations of the CB1R blocker JD-5037. Expression of *CNR1* (encoding for CB1R) was ablated using CRISPR/Cas9 technology. Viability, intracellular stress and signalling were assayed by live-cell probing and real-time PCR. The islet function measured as glucose-stimulated insulin secretion was determined in a perifusion system. Infiltration of immune cells into the islets was monitored by microscopy. Non-obese diabetic mice aged 7 weeks were treated for 1 week with JD-5037, then euthanised. Profiling of immune cells infiltrated in the islets was performed by flow cytometry.

**Results:**

*CNR1* expression was upregulated in circulating CD4^+^ T cells from individuals at type 1 diabetes onset (6.9-fold higher vs healthy individuals) and in sorted islet beta cells from donors with type 1 diabetes (3.6-fold higher vs healthy counterparts). The peripherally restricted CB1R inverse agonist JD-5037 arrested the initiation of insulitis in humans and mice. Mechanistically, CB1R blockade prevented islet NO production and ameliorated the ATF6 arm of the unfolded protein response. Consequently, cyto/chemokine expression decreased in human islets, leading to sustained islet cell viability and function.

**Conclusions/interpretation:**

These results suggest that CB1R could be an interesting target for type 1 diabetes while highlighting the regulatory mechanisms of insulitis. Moreover, these findings may apply to type 2 diabetes where islet inflammation is also a pathophysiological factor.

**Data availability:**

Transcriptomic analysis of sorted human beta cells are from Gene Expression Omnibus database, accession no. GSE121863, available at https://www.ncbi.nlm.nih.gov/geo/query/acc.cgi?acc=GSM3448161.

**Graphical Abstract:**

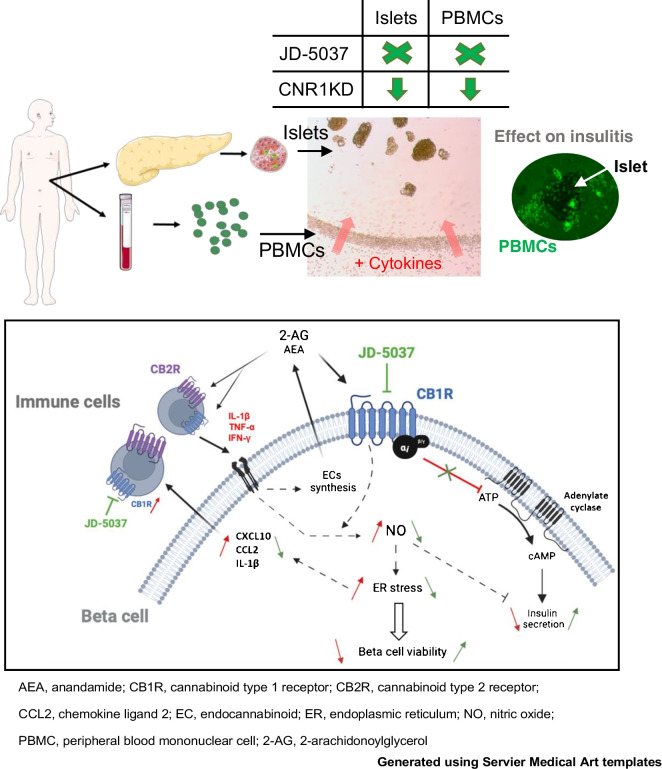

**Supplementary Information:**

The online version of this article (10.1007/s00125-024-06193-6) contains peer-reviewed but unedited supplementary material.


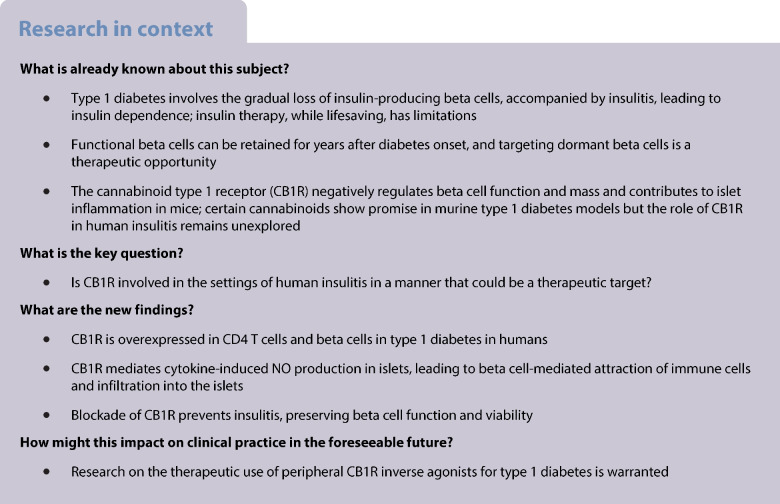


## Introduction

Autoimmune type 1 diabetes is characterised by the progressive loss of insulin-producing beta cell mass and, eventually, insufficient insulin to maintain blood glucose levels within normal ranges. This progressive loss of functional beta cells is preceded by the infiltration of immune T cells in and around islets of Langerhans, an inflammatory process called insulitis [1]. Type 1 diabetes is associated with the production of autoantibodies against beta cell-derived antigens and its aetiology is a mix of genetic and environmental factors. Individuals with type 1 diabetes require exogenous insulin to maintain blood glucose levels within the normal range. However, insulin therapy is linked to dangerous episodes of hypoglycaemia and is often insufficient to control blood glucose levels. At the onset, when clinical manifestations occur (hyperglycaemia, hypo-insulinaemia and the presence of circulating autoantibodies), individuals with type 1 diabetes still have some remaining functioning beta cells that persist for years after hyperglycaemia onset [2, 3]. In individuals with long-standing type 1 diabetes (medalists, over 50 years), residual C-peptide secretion and insulin-positive beta cells are still detectable [4]. Hence, dormant beta cells are a potential therapeutic target to delay the progression of type 1 diabetes and its associated complications. For instance, endogenous insulin secretion is beneficial in controlling glucagon secretion.

The endocannabinoid system (ECS) regulates whole-body metabolism and inflammation. Within the ECS, the cannabinoid type 2 receptor (CB2R) is present in the immune system, while the cannabinoid type 1 receptor (CB1R), present in the central nervous system as well as in peripheral tissues, is expressed in immune cells and islets of Langerhans, particularly endocrine beta cells in mouse and human [5, 6]. The endogenous ligands of the cannabinoid receptors (endocannabinoids) are anandamide (AEA) and 2-arachidonoylglycerol (2-AG), which are synthesised and secreted on demand. Beta cells express the enzymes for their synthesis and degradation (*N*-acyl phosphatidylethanolamine phospholipase D [NAPE-PLD], fatty acid amide hydrolase [FAAH]), diacylglycerol lipase [DAGL] and monoacylglycerol lipase [MAGL]), and synthesise and secrete endogenous ligands, mainly 2-AG, in response to glucose and depolarisation [6].

We have previously described that the CB1R negatively regulates beta cell function and viability in murine models of obesity and its ablation specifically in beta cells in vivo in mice is sufficient to prevent the infiltration of immune cells into the islets [7–9]. In addition, CB1R also plays a role in the islet inflammation driven by immune cells [10]. Certain phytocannabinoids and synthetic cannabinoids have therapeutic properties in preclinical murine models of type 1 diabetes [11–14], through preventing beta cell cytotoxicity and reducing the severity of insulitis. However, the therapeutic potency of targeting CB1R and the involvement of the ECS in insulitis has not been explored. Herein, we examine the role of CB1R in the process of insulitis in humans using a novel ex vivo model of human insulitis, and investigate the therapeutic potential of a peripherally restricted CB1R inverse agonist JD-5037.

## Methods

### Materials

JD-5037 was purchased from Med Chem Express (MedChemtronica, Sweden). Arachidonoyl 2-chloroethylamide (ACEA) was purchased from Tocris (Biogen, Madrid, Spain). Antibodies PE-CyTM7 Rat Anti-Mouse CD8a, PerCP-CyTM5.5 Rat Anti-Mouse CD3 Molecular Complex and APC-CyTM7 Rat Anti-Mouse IFN-γ were obtained from BD Pharmigen (BD Biosciences, Madrid, Spain). Monoclonal CD4 Antibody (GK1.5) and APC were purchased from eBioscienceTM (Invitrogen, Madrid, Spain).

### Ethics

The living human donor protocol followed the principles established in the Declaration of Helsinki of 1964, the World Medical Association and the European Commission agreement of 1996 related to Human Rights and Biomedicine and was previously approved by the Ethics Committee of Cordoba and authorised by the government of Andalucía (1673-N-18, project no. PI-0318–2018, 14 March 2019). All donors were informed and recruited by a medical doctor from the Diabetes Unit of the Regional University Hospital of Malaga. Written informed consent was received before participation. Fully anonymised datasets were processed following EU data protection laws and regulations. The study with human samples was performed according to the Declaration of Helsinki and the Declaration of Istanbul. Islet isolation centres had permission to use islets for scientific research if they were insufficient for clinical transplantation following national regulations and ethical requirements and institutional approvals from the University of Lille (see Human Islets checklist in the electronic supplementary material [ESM]). No organs were procured from prisoners. As the French Biomedical Agency regulates the allocation system in France, every organ was allocated by the Agency in Lille, France (Centre Hospitalier Universitaire, Lille). The ethical committee was bypassed, according to French laws and the local institutional review board (Centre Hospitalier Universitaire, Lille) [15], as the study was monocentric and observational. Informed consent was obtained from all donors. Peripheral blood mononuclear cells (PBMCs) were also isolated from living donors’ blood from the French blood bank (EFS)-Inserm under the ethical agreement PLER/2021/005, for experiments with immune cells only.

Animal care and experimental procedures were approved by the Animal Experimentation Ethics Committee of Malaga University and authorised by the government of Andalucía, Spain (project no. 28/06/2018/107). The European directive 2010/63/EU on the use of animals for research purposes was followed as well as the ARRIVE 2.0 guidelines on reporting experiments involving animals or animal tissues.

### Animals

The sample size was calculated using the G*Power program (α=0.05 and 1 − β=0.95). Mice were housed in groups of four using 12 h dark–light cycles and provided with regular chow (SAFE A04, Panlab) and water ad libitum. Female NOD/ShiLtJ mice develop autoimmune type 1 diabetes spontaneously early in life and are a model for type 1 diabetes, while male NOD/ShiLtJ mice develop autoimmune diabetes late in life as a mature adult (see data at The Jackson Laboratory Website Strain no. 001976; https://www.jax.org/strain/001976). NOD/ShiLtJ female mice (JAX Mice Strain no 001976, commonly called NOD; RRID:IMSR_JAX:001976), 5 weeks old, were purchased from Charles River (Italy; https://www.criver.com/products-services/find-model/jax-nod-mice?region=29). Mice were acclimated for 2 weeks and simultaneously randomised to two groups following simple randomisation: vehicle or 3 mg/kg of JD-5037. After the acclimatisation period, mice (7 weeks old) were given a daily i.p. injection of vehicle or JD-5037 for 1 week. Non-fasting blood glucose was monitored daily from tail bleeds using the OneTouch Ultra blood glucose meter (LifeScan IP Holdings). After a week of treatment, glucose tolerance was determined by an IPGTT. The normal threshold was established using 4-week-old NOD mice (when there was still no insulitis). At the end of the study, mice were euthanised by cervical dislocation and the pancreas was dissected for analysis.

### IPGTT

NOD mice were fasted overnight (16 h) and given water ad libitum before an i.p. injection with a bolus of 2 g/kg of glucose. Blood glucose from tail bleeds was analysed at 0, 15, 30, 60 and 90 min after administration of the bolus.

### Flow cytometry of infiltrated immune cells into the islets

After treatment, mice were killed and the pancreas was dissected. Pancreatic lymph nodes were removed by dissection before pancreas disaggregation. Islets were isolated as described previously [7] by pancreas disaggregation with an infusion of 0.7 mg/ml collagenase P containing DNAse I in HBSS without Ca^2+^/Mg^2+^ following a 9 min incubation at 37°C. The enzymatic reaction was quenched with cold HBSS containing 1% (vol./vol.) horse serum. Whole islets were disaggregated mechanically and filtered with a cell strainer (Corning, Biomol, Seville, Spain). Cells were washed in media and recovered by centrifugation. Erythrocytes were lysed with ACK lysis buffer (Gibco, Thermofisher Scientific, Biomol). Cells were plated in 96-well plates at 2 × 10^5^ cells/well. Cells were washed with FACS buffer and blocked with 0.1% (vol./vol.) goat serum before staining. Corresponding Ig isotype controls were used. Populations were determined by using BD FACS Canto II with FACS Diva and FlowJo software version 10.6.1 (https://www.flowjo.com/solutions/flowjo/downloads) (BD Biosciences).

### Human samples

Blood samples from donors were obtained from the Diabetes Unit of the Hospital Regional Universitario de Malaga, or from volunteers. The sample size was calculated based on a comparative analysis between the study groups (healthy control group and group of individuals at the onset of type 1 diabetes). Based on the mean expression levels and SD obtained previously in associated pathologies for *Cnr1* compared with healthy individuals [5], with a CI of 95% and a statistical power of 80%, the sample size was 11 per group. Both sexes, males and females, were considered without any discrimination. Sex of participants/donors was self-reported. Data on ethnicity and socioeconomic factors were not collected. Inclusion criteria for donors with type 1 diabetes were as follows: regular follow-up in the Diabetes Unit of the Regional University Hospital of Malaga with a recent history of type 1 diabetes (less than 8 months from diagnosis); 18–65 years of age; HbA_1c_ ≤86 mmol/mol (10%); and normal body weight defined as BMI ≥18.5 or ≤24.9 kg/m^2^. Inclusion criteria for healthy donors were as follows: not presenting with type 1 diabetes or having relatives with type 1 diabetes; HbA_1c_ ≤39 mmol/mol (5.7%); and matched in age (33.5 ± 2.5 years), sex and BMI (23.25 ± 0.15 kg/m^2^) to the group with type 1 diabetes. Exclusion criteria were diagnosis of an autoimmune or inflammatory disease distinct from type 1 diabetes, diagnosis of type 2 diabetes or insulin resistance.

### PBMCs and subpopulation isolation

Human PBMCs were isolated from fresh donor blood samples by gradient centrifugation using Ficoll-Paque (GE Healthcare, Madrid, Spain) following the manufacturer’s instructions. Human CD4^+^ T cells were obtained by negative isolation using the EasySep Direct Human CD4^+^ T Cell Isolation Kit (STEMCELL Technologies, Madrid, Spain) following the manufacturer’s instructions. PBMCs and CD4^+^ T cells were spun and frozen at −80°C for later RNA isolation.

### 3D-culture of human islets and PBMCs

Freshly isolated islets (100 islets equivalent [IEQ]) of at least 85% purity were seeded in 1:1 CMRL media and Corning Matrigel Growth Factor Reduced (GFR) Basement Membrane Matrix, Phenol Red-free, lactose dehydrogenase elevating virus (LDEV)-free (henceforward referred to as Matrigel; Cultek SLU, Madrid, Spain) at 2 IEQ/μl. PBMCs isolated from the same donor were stained using CellTrace CFSE (Thermofisher) following the manufacturer’s instructions. Briefly, cells were stained for 20 min at 37°C in 1:1000 CellTrace CFSE in 1× PBS protected from light, following incubation for 5 min in complete media. Cells were then washed and incubated for 10 min in complete media before adding them (in CMRL media) to the 3D-cultured islets in suspension at 10^6^ cells/ml. Co-cultures were insulted with 1000 U/ml of IFN-γ, 100 U/ml of TNF-α and 150 U/ml of IL-1β (Sigma-Aldrich, Biomol), as described previously [16, 17]. Infiltration of immune cells was determined by microscopy.

### Mitochondrial superoxide and nitric oxide live-cells assays

Production of mitochondrial superoxide and NO in islets was determined using the MitoSOX Red Live-cells Assay and the DAF-FM diacetate oxidative stress assay (M-36008 and D-23844, respectively; Invitrogen-ThermoFisher) following manufacturer’s guidelines. In brief, MitoSOX reagent or DAF-FM reagent were added to the media at 1:1000 and incubated for 10 min and 1 h, respectively, at 37°C protected from light. Cultures were carefully washed with 1× PBS and fresh media was added. Images were taken at excitation/emission of 396/620 or 495/515 nm using an inverted microscope (Nikon Eclipse Ti) with NIS-Elements AR 3.0 imaging software (Nikon Europe, Amstelveen, the Netherlands). Images were analysed by densitometry using Image-J 1.52p (Wayne Rasband, NIH, USA; http://imagej.nih.gov/ij).

### Caspase 3 assay in human islets

Intracellular islet caspase-3 cleavage was determined using the Caspase-3 cleavage assay (catalogue no. Ab32042, Abcam, Biomol). Caspase-3 cleavage assay reagents were added to the cultures at 1:2 and incubated for 5 min in the orbital shaker at 500 rev/min and for 30 min at 37°C. Luminescence was measured in a luminometer (Mithras LB 940).

### Cytotoxicity assay in human islets

Cytokine cytotoxicity was determined using the MultiTox-Fluor Multiplex cytotoxicity assay (no. G9200, Promega, Biomol). The diluted AAF reagent (1:1000; Promega) was added at 1:2 to the cultures and incubated for 5 min in the orbital shaker and for 30 min at 37°C. Fluorescence was measured at 485/520 nm in a fluorometer (Mithras LB 940).

### Human islets and PBMCs gene editing

The gRNA targeting *CNR1* was designed using Zhang’s lab online tools (https://www.zlab.bio/resources, accessed 13 May 2024. A single-stranded DNA (ssDNA) containing the 21-nt *CNR1* target sequence was cloned into the pX601-saCas9-mCherry vector (Addgene), which had been previously digested with BsaI, using NEBuilder HiFi DNA Assembly kit (New England Biolabs) according to the manufacturer’s instructions. Positive clones were validated by Sanger sequencing. HEK293T cells were co-transfected with pAAV-2/8, pAD-Δf6 (Addgene), and either pX601-saCas9-mCherry-gRNA-*CNR1* or pX601-saCas9-mCherry (control) to generate Adeno-associated viruses (AAVs) targeting the *CNR1* gene or the control, respectively. AAVs were produced and purified using chloroform extraction and Amicon Ultra Centrifugal Filter Columns (Merck, Millipore). Viral titration was determined by quantitative PCR following the methods described in [18]. Human islets were transduced with 100,000 physical particles/IEQ in complete CMRL media as described before [19], and PBMCs were transfected with pX601_saCas9-mCherry_gRNA-*CNR1* using Lipofectamine 3000 Reagent (ThermoFisher Scientific) following the manufacturer’s instructions. The efficacy and efficiency of CRISPR/Cas9 were controlled by sequencing of the *CNR1* gene followed by TIDE software (https://tide.nki.nl/, accessed 13 May 2024) analyses, and real-time PCR of *CNR1*.

### RNA extraction and real-time PCR

Total RNA from immune cells or islets (500 IEQ) was extracted using RNeasy Micro or Mini Kit (Qiagen), respectively, following the manufacturer’s instructions. Samples were treated with DNase I using the RNase-Free DNase Set (Qiagen) and quantified with the Nanodrop (Thermofisher Scientific). Reverse transcription was performed using the SuperScript IV First-Strand Synthesis System (ThermoFisher Scientific). Gene expression was assayed by quantitative real-time PCR with SYBR Green (BioRad) or TaqMan Gene Expression Master Mix (ThermoFisher Scientific). Primers for SYBR Green PCR were from Eurofins Scientific (sequences are provided in ESM Table 1). Custom FAM dye-labelled TaqMan (Thermofisher Scientific) primers and probes for the distinct isoforms of *CNR1* were from [5]. Predesigned FAM ^TM^ dye -labelled TaqMan primers and probes were purchased from ThermoFisher Scientific. Expression values were corrected by the housekeeping genes *ACTB* and *GAPDH* for immune cells, and *RPLP0* for islets.

### Human chemokine secretion by ELISA

Secretion of the chemokine C-X-C motif chemokine ligand 10 (CXCL10) by human islets or PBMCs was determined in the media using the Human CXCL10/IP-10 Quantikine ELISA Kit from the R&D System (Biotechne, France) following the manufacturer’s instructions. Samples were diluted 1:30 except for control conditions that were 1:1. Plates were analysed in a microplate reader (Multiskan Go from Thermo Scientific) at 450 nm and 540 nm. CXCL10 concentration was calculated using a log/log curve-fit of the absorbance at 450 nm after subtraction of the absorbance at 540 nm.

### Dynamic islet glucose-stimulated insulin secretion

Dynamic glucose-stimulated insulin secretion (GSIS) in human islets freshly isolated from brain-dead organ donors was determined using a two-chamber perifusion system as described previously [20]. After 24 h of culture, an equal amount of 400 IEQ of islets was deposited in the chambers for all conditions. Islets were first perifused for 50 min with a low glucose solution (3 mmol/l) using KRB (in mmol/l: 124 NaCl, 4.8 KCl, 2.5 CaCl_2_^.^H_2_O, 1.2 MgCl_2_^.^6H_2_O, 20 NaHCO_3_ and 0.1% (wt/vol.) BSA, pH 7.3, saturated with 95% O_2_/5% CO_2_ throughout the procedure) at 37°C to equilibrate islets in the perifusion system to basal insulin secretion. The eluate was not collected during this phase. The islets were then perifused for 10 min with non-stimulatory glucose concentration (3 mmol/l), followed by 40 min at 15 mmol/l glucose and then 20 min at low glucose solution (3 mmol/l). Fractions were collected every 2 min at a mean flow rate of 1.00 ml/min for a duration of 70 min. After perifusion, islets were collected and intracellular insulin was extracted by acid–ethanol. Insulin was quantified using a Beckman Coulter Access 2 Analyzer Immunoassay System, and insulin secretion was expressed as total intracellular content normalised to baseline. Stimulation index was calculated as the first-phase GSIS (minutes 14-22)/baseline (S1/B).

### Endocannabinoid extraction and measurement by LC-MS/MS

Determination of endocannabinoids in the media was performed as previously described in [21]. In brief, media proteins were first precipitated with ice-cold acetone and Tris buffer (50 mmol/l, pH 8.0). Next, an ice-cold extraction buffer (1:1 MeOH–Tris buffer + an internal standard [d_4_-AEA]) was added to the samples. Homogenates were then extracted using ice-cold 2:1 CHCl_3_–MeOH and then washed with ice-cold chloroform three times. The samples were then dried under nitrogen and reconstituted in MeOH. Analysis by LC-MS/MS was conducted on an AB Sciex (Framingham, MA, USA) QTRAP 6500+ mass spectrometer coupled with a Shimadzu (Kyoto, Japan) UHPLC System. Liquid chromatographic separation was obtained using 5 μl injections of samples onto a Kinetex 2.6 µm C18 (100 × 2.1 mm) column from Phenomenex (Torrance, CA, USA). The autosampler was set at 4°C and the column was maintained at 40°C during the entire analysis. Gradient elution mobile phases consisted of 0.1% formic acid in water (phase A) and 0.1% formic acid in acetonitrile (phase B). Endocannabinoids were detected in a positive ion mode using electron spray ionisation and the multiple reaction monitoring modes of acquisition, using d_4_-AEA as an internal standard. The collision energy, declustering potential, and collision cell exit potential for the monitored transitions are given in ESM Table 2. The levels of the endocannabinoids (2-AG and AEA) in samples were measured against standard curves.

### Blinding

Treatment of mice and sample collection were not blinded as they were performed by one single investigator. Mouse and human samples were coded and experimental measurements and analysis blinded during the assessment and data analysis process, which was performed by two other investigators. Islet experiments were performed by at least two different investigators, one of them being blinded for the experimental measurement for GSIS.

### Statistics

Statistical analysis was performed using GraphPad Prism version 6.07 (GraphPad Software, Boston, MA, USA; https://www.graphpad.com/). The normal distribution of data was assessed by normality tests. Mean values were compared using Student’s *t* test or Mann–Whitney test for two groups comparisons, and ANOVA with Tukey’s or Dunn’s test for multiple comparisons, for the parametric or non-parametric test, respectively. A *p* value of < 0.05 was considered significant.

## Results

### The expression of CNR1 is increased in CD4^+^ T cells and islets from donors with type 1 diabetes

**Fig. 1:**
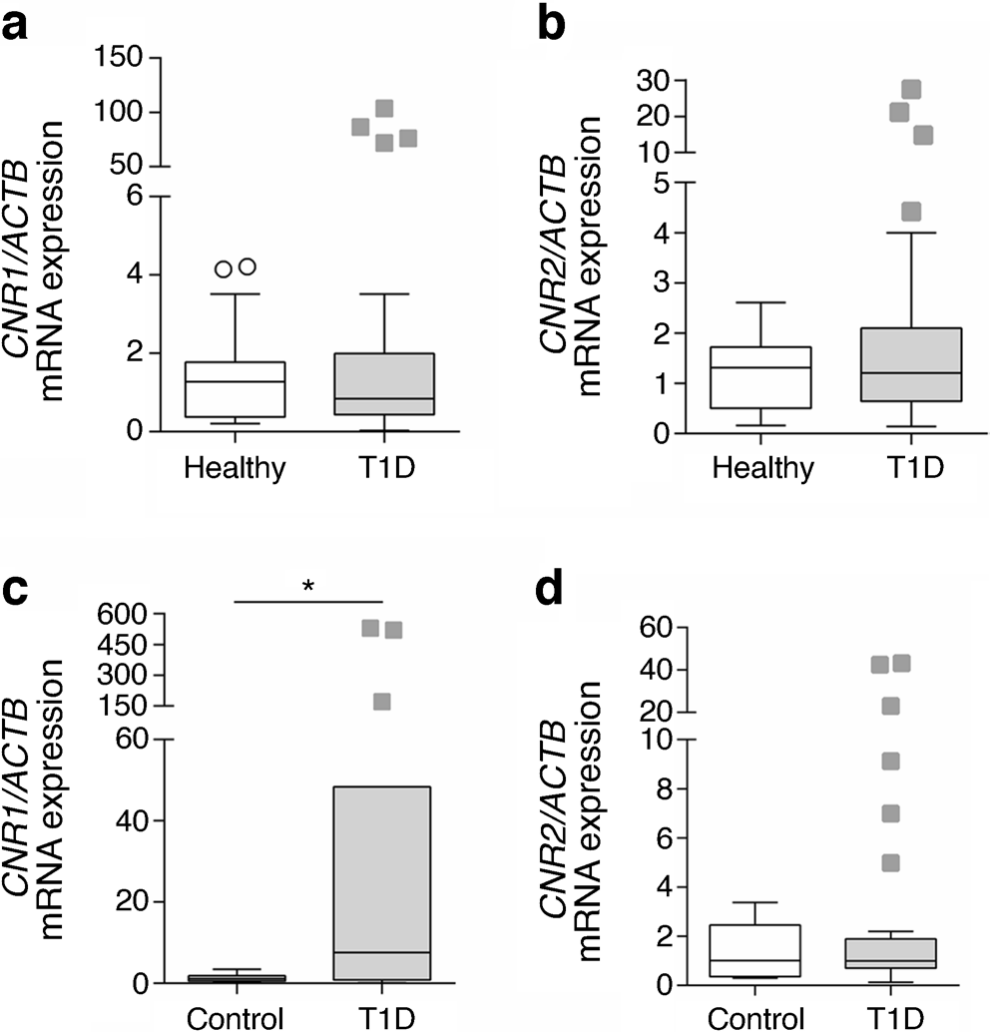
Circulating CD4^+^ T cells have higher *CNR1* expression levels at type 1 diabetes onset compared with levels in healthy donors. Expression of *CNR1* (**a**, **c**) and *CNR2* (**b**, **d**) mRNA in PBMCs (**a**, **b**) and circulating CD4^+^ T cells (**c**, **d**) from the blood of healthy donors (control; *n*=11) and donors with recent onset of type 1 diabetes (*n*=22). The expression of *ACTB* was used as a control. The box and whiskers graphs (Tukey) show data distribution (top and bottom quartiles in boxes and the minimum and maximum value with lines), median and the outliers as single data points outside the box. *p<0.05 (by Mann–Whitney *U* test). T1D, type 1 diabetes

CB1R is expressed in immune cells, and the ECS is overactivated systemically in other pathologies, including type 2 diabetes and autoimmune disorders [22–25]. To investigate the immune cell pathophysiological component, we analysed circulating immune cells from individuals at the onset of type 1 diabetes. Individuals recently diagnosed with type 1 diabetes at the Diabetes Unit of the Regional University Hospital of Malaga, and sex-, age- and BMI-matched healthy donors, were recruited (Table 1) and blood samples were drawn. Thirty three percent of the donors were female and 66% were male, with a median BMI of 23 (range 18–29) kg/m^2^. Donors with a diagnosis of type 1 diabetes showed cardinal signs of diabetes (polyuria, polydipsia and polyphagia) and had an HbA_1c_ of 86 ± 9 mmol/mol (10 ± 3%). Thirty-two percent of the donors with type 1 diabetes were positive for IA2 and 64% were positive for GAD65 antibodies; none were anti-insulin positive. All donors with type 1 diabetes had detectable C-peptide, with a median level of 0.53 (range 0.1–2.1) ng/ml. The expression of *CNR1* and *CNR2* (encoding for CB1R and CB2R, respectively) was analysed by real-time PCR in PBMCs and circulating CD4^+^ T cells from donors. As previously described, both PBMCs and CD4^+^ T cells showed higher expression levels of *CNR2* than of *CNR1* (ESM Fig. 1a). There were no significant differences in the expression of *CNR1* or *CNR2* when comparing PBMCs from donors with vs without type 1 diabetes (Fig. 1a,b). Circulating CD4^+^ T cells from donors with type 1 diabetes displayed an increase of 57-fold for mean and 6.9-fold for median in the expression of *CNR1* (Fig. 1c) but no difference in *CNR2* (Fig. 1d), when compared with cells from non-diabetic donors.

**Table 1:** Donor characteristics

Characteristic	Type 1 diabetes (*n*=22)	Healthy (*n*=11)	*p* value
Age, years	31 ± 13	36 ± 7	0.0982
Sex, % male/% female	55/45	75/25	0.1345
BMI, kg/m^2^	23.1 ± 3.2	23.4 ± 3.6	>0.9999
HbA_1c_, mmol/mol	86 ± 9	<38	0.0043
HbA_1c_, %	10 ± 3	<5.6	0.0043
Duration of diabetes, months	3.7 ± 4.7	<0	0.4154
Cardinal signs of diabetes^a^, %	100	0	<0.0001

Data are mean ± SEM or %

^a^Polyuria, polydipsia and polyphagia

Within the islet microenvironment, besides being expressed in resident/infiltrated immune cells, CB1R is expressed in beta cells [5, 10]. Data mining of transcriptomic analysis of sorted human beta cells from cadaveric donors with or without type 1 diabetes (GSE121863, [26]) revealed that the level of *CNR1* transcripts, but not *CNR2*, is 3.6-fold higher in beta cells from donors with type 1 diabetes than in those from donors without type 1 diabetes (ESM Fig. 1b). Beta cells from type 1 diabetes donors had higher *MGLL* expression levels (encoding the endocannabinoid enzyme MAGL) than those from healthy donors (ESM Fig. 1c). No other changes in ECS variables were found in this cohort (ESM Fig. 1c).

### Pharmacological blockade of CB1R prevents insulitis progression in humans

**Fig. 2:**
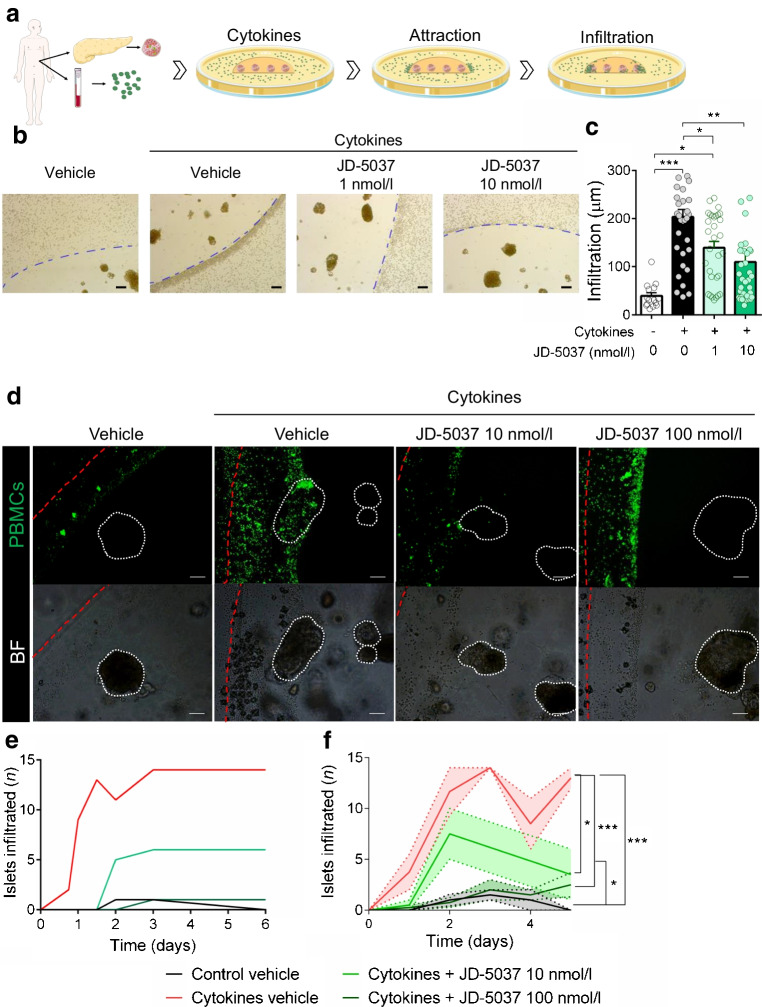
Pharmacological blockade of CB1R by JD-5037 arrests insulitis in an ex vivo human model. (**a**) Schematic representation of the experimental ex vivo human insulitis model (created using Servier Medical Art templates). Human islets and PBMCs were freshly isolated from the pancreas and blood, respectively, of a deceased organ donor. Islets were embedded in Matrigel to create a 3D culture. PBMCs were stained with 488 CellTrace CFSE before adding them to the media in co-culture with the islets. Co-cultures were treated with a mix of cytokines (IFN-γ, IL1-β and TNF-α) and monitored over 6 days. Within the first 18 h, there was chemoattraction of the immune cells towards the border of the 3D culture, followed by their infiltration through the Matrigel towards the islets. (**b**, **c**) Representative photomicrograph (**b**) and quantification (**c**) of the chemoattraction in 3D co-cultures treated with vehicle or increasing concentrations of JD-5037. (**d**) Illustrative photomicrograph after 6 days of insult of the PBMCs (green) and bright field. The border of the Matrigel is delineated with a dotted red line. The islets are delineated with a dotted white line. (**e**, **f**) Quantification of islet infiltration in a single donor (same donor as the photomicrographs) (**e**) and three donors (**f**). Data are mean ± SEM, *n*=3 donors. *p<0.05, **p<0.01 and ***p<0.001 (by one-way [**c**] or two-way ANOVA [**f**]; Tukey post hoc test). Scale bar, 200 μm. BF, bright field

In light of a dysregulation in the ECS occurring in type 1 diabetes, with increased expression of CB1R in immune cells and beta cells, we investigated its role in insulitis. Since CB1R, even in the absence of ligand, exhibits a basal activity [27], we approach its study by using a pharmacological inhibitor, a potent CB1R-specific inverse agonist JD-5037, that would allow inhibition of both basal and agonist-induced activity (i.e. endocannabinoids secreted within the culture). JD-5037 is a synthetic compound with low brain barrier permeability, hence peripherally restricted, with no known off-targets [10, 28–30]. JD-5037 also has a higher affinity for a shorter isoform of the CB1R, CB1b, highly expressed in beta cells [5]. We chose JD-5037 because of these characteristics and because it has been extensively studied in multiple in vitro and in vivo studies, showing therapeutic properties in various murine pathological models [10, 28–30]. We first developed a novel ex vivo model of human insulitis. We obtained human pancreas and blood samples from the same organ donor, and islets and PBMCs were isolated simultaneously. Freshly isolated islets were 3D cultured using Matrigel, in co-culture with the same donor PBMCs in suspension. A mix of cytokines (IFN-γ, IL-1β and TNF-α) was then introduced in the presence of increasing concentrations of JD-5037 or vehicle (Fig. 2a). Dosages of JD-5037 were selected from previous publications and also from our previous experience working with this compound in human islets and were five times lower than the concentrations found in the blood after a maximal non-observed-adverse effect dose was given to mammals [5, 29, 31]. Cytokine treatment induced prompt changes in the ECS, but not long-term changes in endocannabinoid secretion, when comparing the control and cytokine-treated groups (ESM Fig. 2). In particular, treatment with cytokines increased the expression of *DAGLA* and *DAGLB* mRNA within 1 h and *NAPEPLD* (also known as *NAPEPLD*) within 4 h and decreased the expression of *ABHD6* after 24 h (ESM Fig. 2b). The expression of *FAAH* was not detected. Accordingly, secretion of AEA was significantly more augmented in islets treated with cytokines than those treated with vehicle after 24 h (ESM Fig. 2c). Within the first 18 h, immune cells were attracted towards the 3D culture; measurement of cell accumulation at the border of the Matrigel showed that cytokine treatment notably and significantly increased the accumulation of immune cells at the border of the Matrigel compared with control treatment (Fig. 2b,c). Treatment with 1 nmol/l and 10 nmol/l JD-5037 significantly reduced this cytokine-induced accumulation by 31% and 46%, respectively (Fig. 2b,c). We then determined the infiltration of immune cells specifically into the islets in this model by staining PBMCs with a fluorescent cell tracer before co-culturing them with the islets. Cytokines induced the infiltration of immune cells into the islets over time, starting 18 h after their introduction and reaching a plateau after 3 days of culture (Fig. 2d,e). Similar to the previous assay, treatment of the co-culture with JD-5037 (10 nmol/l) significantly decreased the infiltration by 57% compared with vehicle treatment (Fig. 2d,e). A ten-fold higher dose of JD-5037 (100 nmol/l) fully prevented the infiltration of immune cells into the islets cultured in the presence of cytokines for up to 6 days of culture (Fig. 2d–f). The reduction in immune cell infiltration was not associated with CB1R blockade negatively impacting immune cell proliferation, cell viability or the expression of cytokines (ESM Fig. 2d,e). However, JD-5037 prevented cytokine-mediated CXCL-10 secretion from immune cells, an action that may contribute to their activity (ESM Fig. 2f).

These data were validated in vivo in a murine model of autoimmune diabetes, the NOD mouse. In NOD mice, insulitis is known to be triggered at around 4 weeks of age, with infiltration of CD4^+^ T cells. At around 8 weeks of age, cytotoxic T cells (CD8^+^ T cells) then infiltrate, and at around 12 weeks of age beta cell cytotoxicity occurs, eventually leading to hyperglycaemia and onset of type 1 diabetes at around 18 weeks of age (ESM Fig. 3a). We intended to target CB1R after initial infiltration is triggered but before the onset of full insulitis (i.e. 6–7 weeks of age) when activated CD4^+^ T cells are driving the process. Seven-week-old NOD mice were treated daily with 3 mg/kg of JD-5037 i.p. for 1 week and euthanised to isolate the islets and profile the early infiltration of immune cells by flow cytometry. None of the mice were or became dysglycaemic in the course of the experiment (ESM Fig. 3b) and non-fasting blood glucose was 5.9 ± 0.2 and 5.7 ± 0.2 mmol/l in the vehicle and JD-5037 treated groups, respectively. Flow cytometry analysis showed that 1 week of treatment with JD-5037 suspended the progression of infiltration of immune cells into the islets, significantly preventing infiltration of T cells into the pancreas (CD3^+^ cells; ESM Fig. 3c). No changes were observed in CD4^+^ T cells (ESM Fig. 3d,e). However, treatment with JD-5037 significantly reduced Th1 CD4^+^/IFN-γ^+^ T cells (ESM Fig. 3f) without altering other Th cell types such as Th17 CD4^+^/IL-17^+^ T cells (ESM Fig. 3g). JD-5037 also prevented the infiltration of CD8^+^ T cells (ESM Fig. 3h,i). Overall, JD-5037 was able to arrest the polarisation of CD4^+^ towards the Th1 profile and the initiation of infiltration of immune cells into the islets in young NOD mice, possibly due to the blockade of CB1R in the immune cells and/or its blockade in beta cells.

### Blockade of CB1R protects human islets from cytokine-induced cell death and NO production

**Fig. 3:**
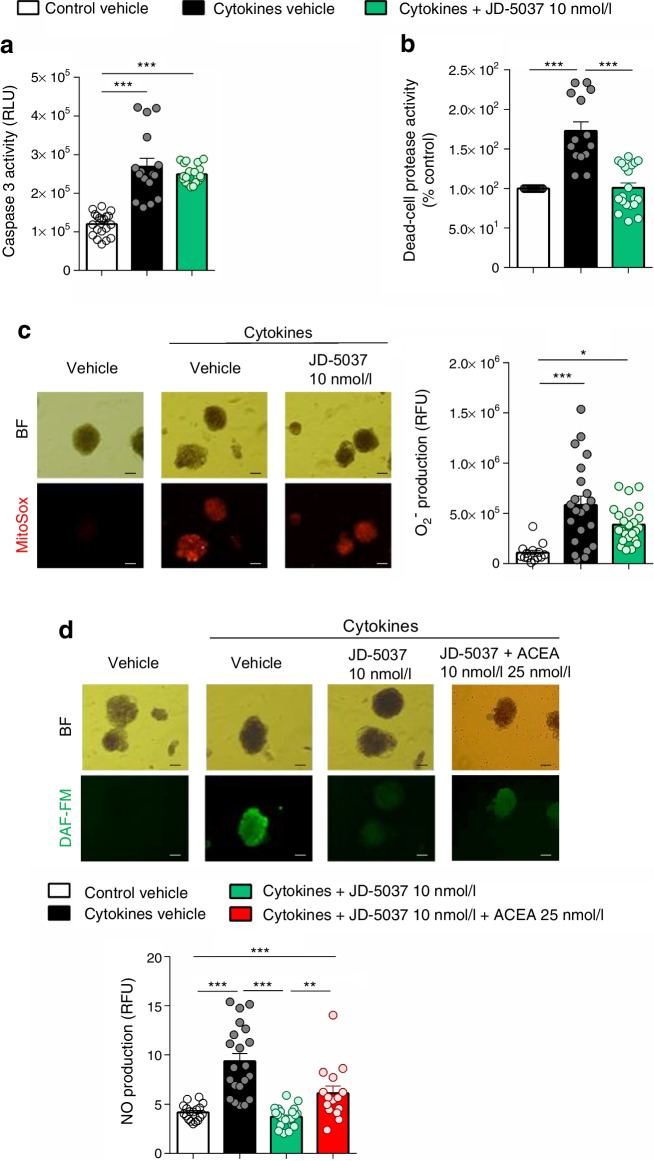
JD-5037 prevents cytokine-induced cell death and nitric oxide (NO) production in human islets ex vivo. (**a**, **b**) Apoptosis and cell death were measured as caspase 3 activity (**a**) and dead-cell protease activity (**b**) in islets stimulated with or without cytokines and treated with vehicle or JD-5037. (**c**, **d**) Representative photomicrographs showing mitochondrial superoxide (O_2_^−^) (**c**) and cellular NO production (**d**) as stained with MitoSox (red) and DAF-FM (green), respectively, in islets stimulated with or without cytokines and treated with vehicle or JD-5037 alone or in combination with ACEA (25 nmol/l). Bright-field photomicrographs and fluorescence imaging of the same field are shown; the graph shows quantification of the relative fluorescence units for MitoSox or DAF-FM staining. Scale bar, 200 μm. Data are mean ± SEM and individual data points, *n*=3 donors; *n*=20–25 islets per group. *p<0.05, **p<0.01 and ***p<0.001 (by one-way ANOVA; Tukey post hoc test). BF, bright field; RFU, relative fluorescence units; RLU, relative light units

We have previously described that genetic ablation of CB1R in beta cells preserves beta cell viability and prevents inflammation in mice subjected to inflammation [7]. We determined cytokine-induced islet cell death and apoptosis 24 h after the insult ex vivo in human islets. Cytokine treatment induced a 1.7-fold and 2.2-fold increase in dead-cell protease and caspase 3 activity, respectively, compared with control treatment (Fig. 3a,b). Treatment with JD-5037 did not affect caspase 3 activity (Fig. 3a) but it did prevent cytokine-induced cell death (Fig. 3b). Beta cells have a low antioxidant response, being specially sensitive to oxidative stressors. Since cytokines trigger the production of reactive oxygen species (ROS) [32], which in turn impacts islet viability, we determined the mitochondrial production of superoxide in living islets. While in the control condition, islet ROS production was negligible; cytokines triggered a 5.3-fold increase in ROS production that was not prevented by JD-5037 treatment (Fig. 3c). Reactive nitrogen species (RNS) have been reported to play an important role in beta cell fate, function and viability and NO mediates intra-islet cytokine signalling pathways [33]. Cytokine treatment induced a 1.9-fold increase in NO production in living islets compared with control vehicle treatment; this response was fully ablated by JD-5037 treatment (Fig. 3d). To confirm the specificity of the effect of JD-5037, we used a potent CB1R agonist, ACEA. ACEA prevented the reduction in NO production induced by JD-5037, with NO levels being significantly higher than those found in JD-5037-treated islets (Fig. 3d), indicating that the effect of JD-5037 is specific to CB1R blockade. ACEA alone did not affect cytokine-mediated islet viability or NO production (ESM Fig. 4a,b), most probably due to the already high levels of endocannabinoids secreted to the media.

**Fig. 4:**
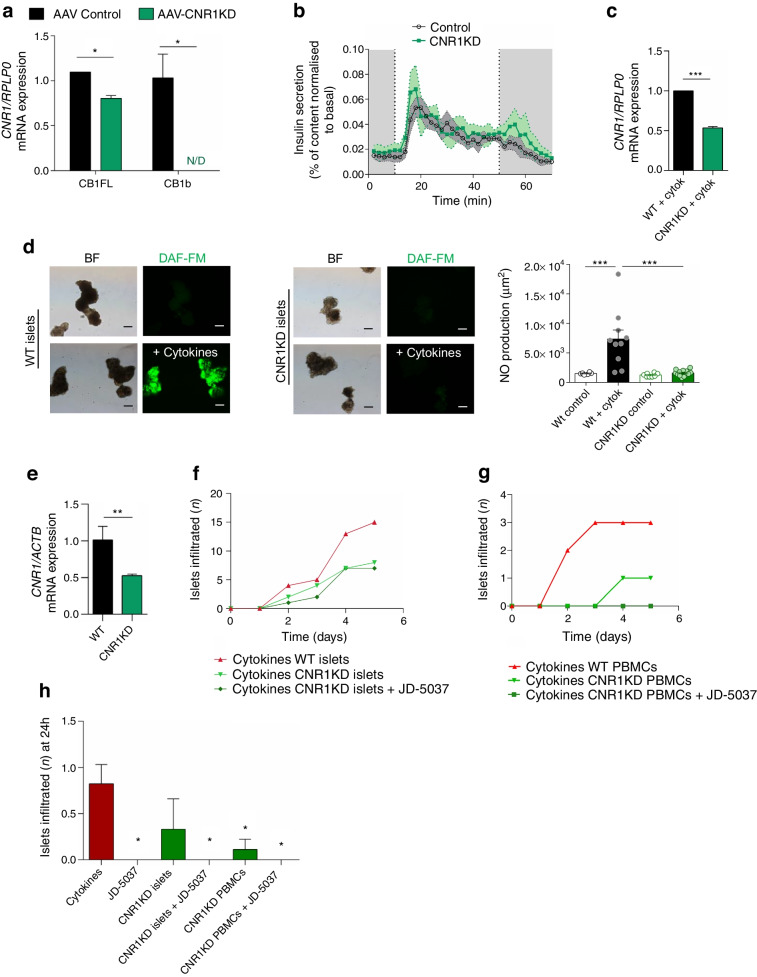
*CNR1* ablation in human islets prevents cytokine-induced NO production and insulitis ex vivo. *CNR1* was knocked down in human islets or PBMCs using CRISPR/Cas9, and cytokine-mediated damage was investigated in the human insulitis ex vivo model. (**a**) mRNA expression of *CNR1* full length (CB1FL) and b isoform (CB1b) in CNR1KD and control islets. (**b**) GSIS in a perifusion system. (**c**) *CNR1* expression after cytokine treatment. (**d**) NO production in wild-type and CNR1KD islets. Representative photomicrographs showing NO production as stained with DAF-FM (green), and bright-field photomicrographs of the same field; the graph shows quantification of the relative fluorescence units for DAF-FM staining. Scale bar, 200 μm. (**e**) mRNA expression of *CNR1* in CNR1KD and wild-type PBMCs. (**f**, **g**) Infiltration of wild-type immune cells in co-culture with wild-type or CNR1KD islets (**f**) and infiltration of wild-type or CNR1KD immune cells in co-culture with wild-type islets (**g**). (**h**) Early infiltration (24 h) of islets in the ex vivo model using CNR1KD islets, and CNR1KD PBMCs with or without JD-5037 (*n*=3 independent experiments). *p<0.05, **p<0.01 and ***p<0.001 (by *t* test or two-way ANOVA; Tukey post hoc test). BF, bright field; WT, wild-type

To investigate the contribution made by CB1R to both axes of insulitis (i.e. immune and beta cells), we next attempted to reduce *CNR1* expression (cannabinoid receptor 1 knockdown [CNR1KD]; Fig. 4) using CRISPR/Cas9 technology. CNR1KD islets showed an adequate GSIS profile, a 20% reduction in *CNR1* full-length expression and full ablation of the main beta cell CB1R isoform [5], the *CNR1* b splice variant (Fig. 4a–c). Cytokines did not induce NO production in CNR1KD islets compared with control islets (Fig. 4d). Both CNR1KD islets and CNR1KD PBMCs (Fig. 4e–g) led to reduced early insulitis in the ex vivo model compared with the control. In both cases, JD-5037 fully prevented early immune cell infiltration (Fig. 4h) but did not affect the progression of insulitis in CNR1KD islets (Fig. 4f). These data show that CB1R in both islets and immune cells contributes to the initiation of insulitis but it could not be determined whether full ablation in beta cells would suffice to fully arrest insulitis, since *CNR1* expression was not completely eliminated by CRISPR/Cas9. However, these data suggest that the progression of insulitis is controlled by CB1R in the islets (Fig. 4f).

### CB1R blockade ameliorates the ATF6 arm of the unfolded protein response to cytokines in human ex vivo islets

**Fig. 5:**
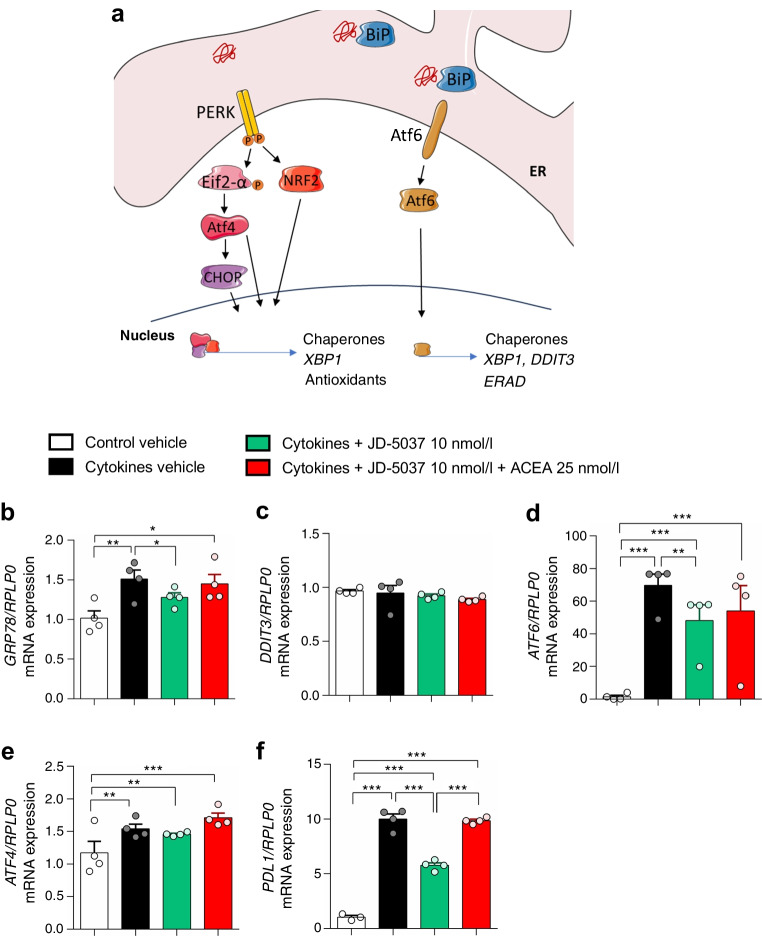
Targeting CB1R modulates ER stress response to cytokines in human islets ex vivo. Human islets were treated with JD-5037 (10 nmol/l), JD-5037 in combination with ACEA (25 nmol/l), or vehicle before an insult with cytokines, and samples were collected at 1 and 4 h for RNA extraction. (**a**) Schematic representation of ER stress signalling (created using Servier Medical Art templates). (**b**–**f**) Expression of *GRP78* (also known as *HSPA5* and *BIP*) (**b**), *DDIT3* (also known as *CHOP*) (**c**), *ATF6* (**d**), *ATF4* (**e**) and *PDL1* (also known as *CD274*) (**f**) after 1h (*GRP78*) and 4 h (the rest) of insult, respectively. The expression of *RPLP0* was used as a control. Data are mean ± SEM, *n*=4 donors. *p<0.05, **p<0.01 and ***p<0.001 (by one-way ANOVA; Tukey post hoc test)

It has been suggested that NO induces beta cell death through the activation of endoplasmic reticulum (ER) stress [34], and ER stress in beta cells precedes type 1 diabetes onset [35]. We investigated the unfolded protein response as a mediator of ER stress in cytokine-treated islets (Fig. 5a). Cytokines induced the expression of *GRP78* (*BIP*, also known as *HSPA5*, encoding for binding-immunoglobulin protein [BiP]) (Fig. 5b). Treatment with JD-5037 alone, and not in combination with ACEA, prevented elevated *GRP78* expression (Fig. 5b). No significant effects on the expression of the gene encoding C/EBP homologous protein (*DDIT3*, also known as *CHOP*) were detected after adding the mix of cytokines (Fig. 5c), as previously reported [36]. The expression of *ATF6*, downstream of BiP, followed the same pattern as *GRP78* (Fig. 5d), while JD-5037 had no impact on cytokine-induced *ATF4* expression (Fig. 5e).

Cytokines and ER stress are reported triggers of *PDL1* (also known as *CD274*) expression in beta cells, which occurs in type 1 diabetes [37]. Non-diabetic beta cells do not express *PDL1*, unlike beta cells from donors with type 1 diabetes, and the expression levels correlate with higher chemokine expression and immune cell–beta cell interaction [37–39]. We found that cytokines induced the expression of *PDL1* in islets (Fig. 5f). Blockade of CB1R ameliorated the expression of *PDL1*, and the amelioration was prevented when ACEA was present (Fig. 5f).

### Blockade of CB1R reduces the expression of cytokines and chemokines in islets in the ex vivo model of human insulitis

**Fig. 6:**
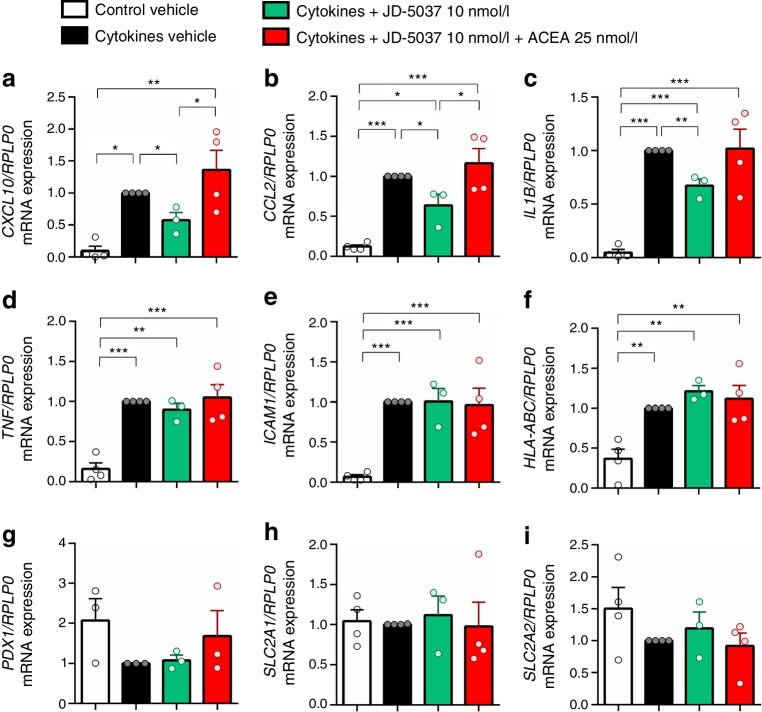
CB1R modulates chemokine expression upon cytokine insult in human islets. Human islets were treated with JD-5037 (10 nmol/l), JD-5037 in combination with ACEA (25 nmol/l), or vehicle before an insult with cytokines, and samples were collected after 24 h for RNA extraction. Expression of *CXCL10* (**a**), *CCL2* (**b**), *IL1B* (**c**), *TNF* (**d**), *ICAM1* (**e**), *HLA-ABC* (**f**), *PDX1* (**g**), *SLC2A1* (**h**) and *SLC2A2* mRNA (**i**) was measured. The expression of *RPLP0* was used as a control. Data are mean ± SEM, *n*=4 donors. *p<0.05, **p<0.01 and ***p<0.001 (by one-way ANOVA; Tukey post hoc test)

We further determined the expression of key components of this proinflammatory process in islets 24 h after insult with the mix of cytokines. Cytokines induced the expression of *CXCL10*, *CCL2*, *IL1B*, *TNF*, *ICAM1* and *HLA-ABC* in islets (Fig. 6a–f). Islets treated with JD-5037 (10 nmol/l) displayed significantly lower expression levels of *CXCL10*, *CCL2* and *IL1B* compared with vehicle-treated islets. Expression data was validated by ELISA. Cytokines significantly increased islet secretion of CXCL10 into the media. CB1R blockade by JD-5037 prevented the increased secretion of CXCL10, while ACEA did not induce a synergistic effect with cytokines (ESM Fig. 4c). The effect of JD-5037 was CB1R-dependent as it was reversed in the presence of ACEA (Fig. 6a–c). Treatment with JD-5037 alone or in combination with ACEA did not modulate the expression of *TNFA*, *ICAM-1* or *HLA-ABC* induced by cytokines (Fig. 6d–f). Within the time frame of this experimental intervention, we found no significant alterations in the expression of *PDX1*, *SLC2A1* (encoding for GLUT1) or *SLC2A2* (encoding for GLUT2) either in cytokine-insulted islets, in agreement with previous transcriptomic data [40], or in islets co-treated with cannabinoids (Fig. 6g–i).

### Blockade of CB1R protects beta cells from cytokine-mediated loss of function

**Fig. 7:**
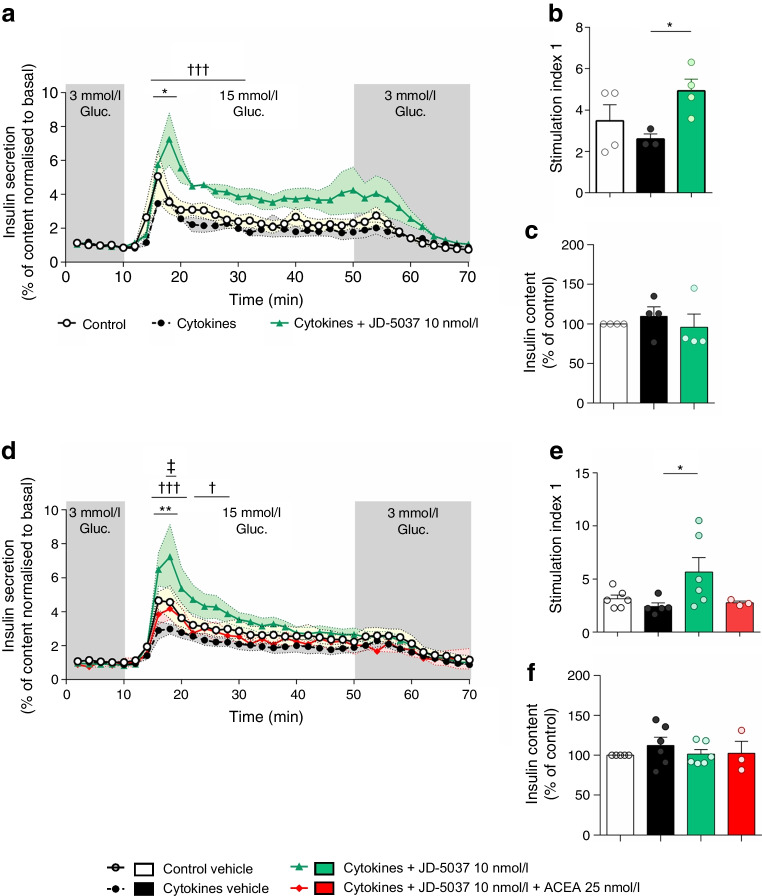
JD-5037 protects beta cell function in the face of inflammation. (**a**–**c**) Dynamic GSIS after 2 h of insult with a mix of cytokines (**a**), stimulation index (**b**) and intra-islet insulin content (**c**) of islets from *n*=4 donors. (**d**–**f**) Dynamic GSIS after 24 h of insult with a mix of cytokines (**d**), stimulation index (**e**) and intra-islet insulin content (**f**) in islets from *n*=6 donors (except for ACEA [*n*=3 donors]). Data are mean ± SEM (shadowed in **a**, **d** or error bars in **b**, **c**, **e**, **f**). *p<0.05 and **p<0.01 for cytokine vs control groups; †*p*<0.05 and †††*p*<0.001 for JD-5037+cytokine vs vehicle+cytokine group; ‡*p*<0.05 for ACEA-JD-5037+cytokine vs JD-5037+cytokine group (by one- or two-way ANOVA; Tukey post hoc test)

We determined beta cell function in islets using a perifusion system to better mimic the physiological secretion of insulin [20]. Human islets were treated with JD-5037 or vehicle for 1 h before adding the cytokine mix. Acute (2 h; Fig. 7a–c) and chronic (24 h; Fig. 7d–f) cytokine treatment induced beta cell dysfunction, with a significant reduction in first-phase insulin secretion (by 1.5-and 1.6-fold, respectively) compared with control, without any change in intracellular insulin content (Fig. 7a–d and ESM Fig. 5). Treatment with JD-5037 prevented the cytokine-induced dysfunction in islets, further enhancing first- and second-phase insulin secretion in both acute and chronic cytokine treatment (Fig. 7a,b,d,e). Beta cell function preservation by JD-5037 was prevented by co-treatment with ACEA, indicating a CB1R-specific role (Fig. 7d,e). Changes in GSIS were not associated with long-term changes in endocannabinoid secretion (ESM Fig. 2).

## Discussion

After hyperglycaemia onset due to type 1 diabetes, some beta cells remain present in islets for many years [4]. Maintenance of the remaining functional beta cell mass at onset and/or recovery of dormant beta cells is a therapeutically targeted window for the treatment of type 1 diabetes. Herein we show, in vivo and ex vivo, in murine and human models, that CB1R plays a key role in the initiation of insulitis and that its pharmacological blockade can ameliorate this pathophysiological process. We further provide convincing data that investigating the potential therapeutic effects of second-generation CB1R inverse agonists is of interest in preventing the progression of insulitis in type 1 diabetes.

Overactivation of the ECS has been described in multiple pathologies including type 2 diabetes [5, 41, 42], for which blockade of CB1R has been explored as a therapeutic strategy [43]. In addition, CB1R may be responsible for CD4^+^ T cell polarisation [23], and changes in circulating CD4^+^ T cells have been described in individuals at the onset of type 1 diabetes [24]. Specifically, polarisation of CD4^+^ T cells to the proinflammatory Th1 phenotype allows the expression of *CNR1* in this cell type [25]. Activation of T cells leads to elevated expression levels of the otherwise almost absent cannabinoid receptor CB1R (eightfold higher than non-activated T cells), without altering the classic immune cannabinoid receptor CB2R [25], enabling the response of T cells to cannabinoids by both receptors. We found, for the first time, that individuals at the onset of type 1 diabetes display increased CB1R expression in circulating CD4^+^ T cells. Islet beta cells from donors with type 1 diabetes also displayed increased expression levels of CB1R. The role of CB1R in the immune cell–beta cell crosstalk at the islet level was previously outlined by us and others in rodent models. Genetic ablation of CB1R specifically in macrophages or in beta cells prevents islet inflammation in diet-induced obesity [7, 10]. Our findings in humans suggest that CB1R plays a role in both compartments (immune cells and islets) in type 1 diabetes. Ingenuity pathway analysis of transcriptomic data obtained from the human pancreas has shown that CB1R is the top upregulator in the period leading up to the onset of type 1 diabetes [44]. The question of whether the increase in CB1R signalling in the pancreas from donors with pre-type 1 diabetes results from beta cells or resident/infiltrated immune cells within the islets remains unanswered but is most likely due to both. Regardless, here we show that cytokines induce dysregulation of the ECS in islets, in keeping with an alteration of the ECS in beta cells of donors with type 1 diabetes. This alteration will alter the homeostatic crosstalk with infiltrating immune cells, sustaining the pathological inflammatory event and further promoting intracellular inflammation and immune cell recruitment. Of note, cytokines induced a reduction in the expression of *ABHD6*, an enzyme that hydrolases 2-AG as well as other lipids and has ECS-independent functions. The alpha/beta-hydrolase domain containing 6 (ABHD6), through the metabolites produced by its activity, regulates other signalling systems, including the inhibition of proinflammatory activity in macrophages [45] and GSIS in beta cells [46]. Therefore, the reduced expression levels of *ABHD6*, besides being responsible for the increase in 2-AG levels, could also represent a compensatory mechanism in response to cytokines.

Previous work by Weiss et al and by us showed that cannabidiol (CBD) and its derivative abn-CBD have the potential to delay and arrest the progression of type 1 diabetes in NOD mice [11–13]. CBD is a pleiotropic cannabinoid, and the implications of CB1R on the effect of CBD or abn-CBD has not been investigated. A (+)-enantiomer of CBD with enhanced binding for the cannabinoid receptors harbouring CB1R antagonism and CB2R agonism activity protected the beta cell mass from streptozocin-induced damage in mice [14]. In the current study, we focused on the specific role of CB1R in insulitis by using JD-5037, which displays enhanced CB1R specificity and inverse agonism activity [28]. When JD-5037 was given to NOD mice in the period leading up to onset of insulitis, it arrested the initiation of insulitis, maintaining the number of cytotoxic T cells at levels similar to those of control non-diabetic mice and reduced the proinflammatory Th1 population compared with vehicle-treated mice. These data highlight an active role of CB1R in the aetiology of insulitis. Additionally, CB1R blockade did not affect Th17, a T cell subset critically involved in autoimmunity. The phenotype observed in vivo could be due to the blockade of CB1R in immune cells or beta cells, or both. Previous studies have shown that the specific CB1R agonist ACEA does not impact immune cell proliferation, which is regulated by CB2R [47, 48]. Similarly, blockade by JD-5037 had no impact on immune cell proliferation or viability. In our human model ex vivo, we show that the blockade of CB1R impacts the crosstalk between islets and immune cells in the pathophysiology of insulitis by preventing the expression of chemokines or cytokines in islets. We further show that CB1R in both immune cells and islets contributes to the initiation of insulitis. Miranda et al, in an in vitro assay, showed that macrophages treated with the CB1R blocker AM251 were able to prevent Th1 T lymphocyte polarisation [49]. We hypothesise that blockade of CB1R in immune cells prevents their proinflammatory activity while its blockade in beta cells prevents intra-islet inflammation, in conjunction leading to negative regulation of insulitis.

In NOD mice [35] and later in humans [50, 51], an active role was ascribed to beta cells in the pathophysiology of type 1 diabetes, via disruption of protein folding and consequent defects on insulin secretion and triggering of ER stress. Modulation of these pathways protects against type 1 diabetes in NOD mice [52, 53]. Mechanistically, we show that CB1R is a negative regulator of cytokine-mediated NO production but not ROS production in human islets. In addition, JD-5037 was able to preserve islet viability and function, highlighting the relevance of NO to beta cell dysfunction. The lack of effect on ROS production agrees with the observation that JD-5037 was not able to prevent the increased caspase 3 activity induced by cytokines. It has also been shown that NO mediates insulin secretory dysfunction in type 1 diabetes [54]. We found that acute and chronic treatment of human islets with cytokines led to beta cell dysfunction, without changes in the expression of GLUT1, GLUT2 and/or PDX1. The preservation of beta cell function by CB1R blockade can therefore be explained by the prevention of the increase in intracellular NO levels. The inhibition of cytokine-mediated NO production, hence ER stress, may result from a direct downregulation of cytokine signalling, as evidenced by reduced activation of signal transducer and activator of transcription 1 (Stat1) and NF-κB in beta cell-specific CB1R knockout islets [7].

A recent study showed that ER stress originates in individuals with insulin resistance associated with beta cell work overload, with concomitant defects in insulin processing and loss of beta cell identity [55]. Our data suggest that, similarly, blockade of CB1R in islets would benefit people with impaired glucose tolerance regardless of its cause because ER stress is prevented. Blockade of CB1R in islets for therapeutic purposes in type 2 diabetes has been explored by us and others; the capability of this blockade in preventing beta cell identity loss has not been deeply explored even though CB1R regulates pancreatic islet microarchitecture during development.

Moreover, we found that the blockade of CB1R not only prevents dysfunction but also further improves the secretory capacity of human islets in a proinflammatory environment when compared with control conditions. Recently, a novel biased CB1R antagonist, MRI-1891, was shown to improve GSIS in INS1 cells in the absence of incretins independently of cAMP [56]. CB1R mainly signals through Gαi, inhibiting adenylyl cyclase activity and cAMP synthesis, but it can also signal through the recruitment of β-arrestin. Indeed MRI-1891, also known as monlunabant, has functional selectivity for β-arrestin [57]. We have previously shown that JD-5037 (non-biased) enhances incretin-mediated glucose-dependent insulin secretion by modulating adenylyl cyclase activity [5, 8]. Timewise, CB1R signals in waves, with a first wave associated with Gαi and a second wave associated with β-arrestin [58]. There is a parallelism of this biphasic response with insulin secretion from beta cells, which also happens in two waves: the first phase corresponds to a peak of insulin within minutes of glucose stimulation, followed by a second phase of insulin being secreted if glucose concentration is maintained. Mechanistically, the first phase occurs from the fusion of granules of insulin close to the membrane, named the readily releasable pool of insulin, while the second phase requires the mobilisation of the reserve pool to replenish the readily releasable pool. Unfortunately, Ghosh et al [56] assessed insulin secretion by static incubation and not in a perifusion system. Whether the biased compound MRI-1891 specifically improves only one phase of insulin secretion or both, hence whether CB1R regulates insulin pulsatility, remains unstudied.

Recently, the anti-CD3 antibody teplizumab has been approved by the US Food and Drug Administration for the treatment of children with a predisposition to type 1 diabetes, as well as individuals with type 1 diabetes [59, 60]. However, disease progression is inexorable and serious side effects can occur during teplizumab treatment. The side effects include decreased lymphocyte count, risk of cytokine release syndrome, and severe life-threatening infections. Other strategies that are safer and more effective at preserving the functionality of beta cells are warranted, especially in children. Synthetic blockers of CB1R have been refined in the last two decades, with second- and third-generation compounds that are more specific, more potent and safer [43]. Some of these novel compounds are in phase 2 clinical trials for related pathologies, including diabetic nephropathy, and are therefore of research interest as potential prophylactics and therapeutics for type 1 diabetes.

The present study has limitations associated with the model used. The human ex vivo insulitis model exploited here allowed for a better understanding of the crosstalk between immune cells and beta cells in the settings of insulitis in humans; however, it does not account for the role that other tissues play in its pathophysiology (e.g. lymph nodes, thymus, gut and the possible impact of the microbiome). While we considered using a complex population of immune cells (i.e. PBMCs) instead of isolated immune cell types (such as CD4^+^ T cells) to account for interactions between distinct immune cell types (such as macrophage-to-T lymphocytes, and others), this approach remains an artificial system that necessitated in vivo validation. Treatment with JD-5037 was able to arrest early insulitis in vivo in mice but it did not impact the Th17 population. Whether long-term CB1R blockade in vivo can influence autoimmunity, T cell maturation or other processes associated with type 1 diabetes development in humans, remains unexplored. Also, we did not analyse and report the data based on sex differences due to the low number of donors. Whether there are sex differences on the role of the ECS in insulitis remains to be investigated.

**Fig. 8:**
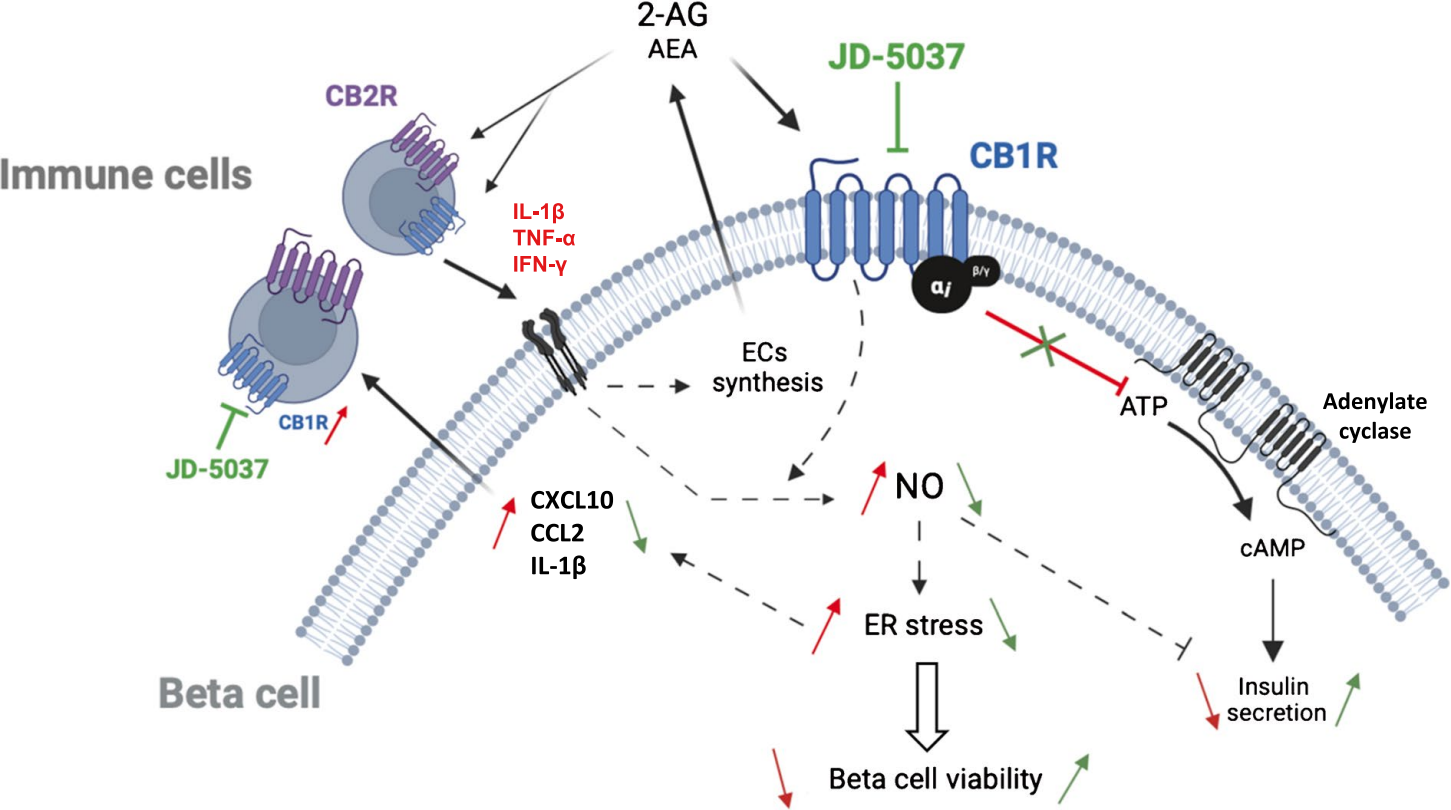
Schematic representation of the regulation of crosstalk between beta cells and immune cells by the ECS. Activated proinflammatory immune cells express CB1R in addition to CB2R. Cytokines secreted by immune cells (or another source) activate their receptors in beta cells, leading to the production of NO and the synthesis and secretion of endocannabinoids (AEA and 2-AG). The endocannabinoids activate the cannabinoid receptors in both beta cells (CB1R) and immune cells (CB1R and CB2R). In beta cells, activation of CB1R further enhances NO–ER stress signalling pathways, leading to chemokine secretion and beta cell death. Chemokines further attract immune cells to the site. Activation of CB1R also leads to a reduction in GSIS. The effect of cytokines is illustrated with red lines/arrows and the effect of JD-5037 with green lines/arrows. CCL2, chemokine ligand 2; EC, endocannabinoid. This figure was created using Servier Medical Art templates

In summary, our findings enhance our understanding of insulitis and the role of the ECS within it. Islets have an autonomous ECS; they synthesise both endocannabinoids which, during GSIS, negatively regulate insulin secretion [6]. However, under conditions of sustained ECS activation, endocannabinoids can become mediators of inflammation. Ex vivo culture of human islets induces stress within them, leading to the eventual secretion of endocannabinoids. The addition of cytokines induces a rapid change in the ECS, resulting in increased islet endocannabinoid levels that peak within 24 h of culture. Hence, the ECS appears to serve as a highly sensitive alert system within the islets, capable of sensing stressors. We theorise that activation of CB1R by secreted endocannabinoids in response to mild stress triggers NO-mediated ER stress as a protective mechanism in beta cells, promoting adaptation and survival [34, 55]. However, persistent CB1R activation in response to chronic stress and inflammation, initially acting as a sensitive defensive mechanism, ultimately contributes to the irreversible loss of beta cells [61, 62] (see Fig. 8), as observed in chronic marihuana consumers (and hence chronic CB1R activation by Δ^9^-tetrahydrocannabinol) [63].

Overall, we described a novel role of the ECS, specifically the CB1R, in the pathophysiology of autoimmune type 1 diabetes. Our study underscores the potential therapeutic promise of the latest generations of CB1R inverse agonists for delaying the progression of insulitis. These findings are particularly relevant, aligning with the recent success of peripheral CB1R inverse agonists in clinical trials aimed at treating metabolic disorders.

## Supplementary Information

Below is the link to the electronic supplementary material.

ESM 1 (PDF 1746 KB)

## Data Availability

Transcriptomic analysis of sorted human beta cells are available at Gene Expression Omnibus database, accession no. GSE121863, available at https://www.ncbi.nlm.nih.gov/geo/query/acc.cgi?acc=GSM3448161. The data from the study in humans are not openly available due to reasons of sensitivity and are available from the corresponding author upon reasonable request. Data are located in controlled access data storage at IBIMA-Plataforma BIONAND and Inserm UMR1190, CHU of Lille.

## Abbreviations

AAV: Adeno-associated virus
ACEA: Arachidonoyl 2-chloroethylamide
AEA: Anandamide
2-AG: 2-Arachidonoylglycerol
BiP: Binding-immunoglobulin protein
CBD: Cannabidiol
CB1R: Cannabinoid type 1 receptor
CB2R: Cannabinoid type 2 receptor
CNR1KD: Cannabinoid receptor 1 knockdown
CXCL10: C-X-C motif chemokine ligand 10
DAGL: Diacylglycerol lipase
ECS: Endocannabinoid system
ER: Endoplasmic reticulum
GSIS: Glucose-stimulated insulin secretion
IEQ: Islets equivalent
MAGL: Monoacylglycerol lipase
PBMC: Peripheral blood mononuclear cell
RNS: Reactive nitrogen species
ROS: Reactive oxygen species
