# Correction: Pharmaceutical targeting of the cannabinoid type 1 receptor impacts the crosstalk between immune cells and islets to reduce insulitis in humans

**DOI:** 10.1007/s00125-025-06372-z

**Published:** 2025-02-15

**Authors:** Elise Wreven, María Soledad Ruiz de Adana, Stéphan Hardivillé, Valery Gmyr, Julie Kerr-Conte, Mikael Chetboun, Gianni Pasquetti, Nathalie Delalleau, Julien Thévenet, Anaïs Coddeville, María José Vallejo Herrera, Liad Hinden, Inmaculada Concepción Benavides Espínola, Mireia Gómez Duro, Lourdes Sanchez Salido, Francisca Linares, Francisco-Javier Bermúdez-Silva, Joseph Tam, Caroline Bonner, Josephine M. Egan, Gabriel Olveira, Natalia Colomo, François Pattou, Isabel González-Mariscal

**Affiliations:** 1 Inserm UMR1190 – Translational Research for Diabetes, Université de Lille, CHU Lille, Institut Pasteur de Lille, Inserm, European Genomic Institute for Diabetes, Lille, France; 2 Servicio de Endocrinología y Nutrición, Hospital Regional Universitario de Málaga, Instituto de Investigación Biomédica de Málaga y Plataforma en Nanomedicina-IBIMA-Plataforma BIONAND, Málaga, Spain; 3 Centro de Investigación Biomédica en Red de Diabetes y Enfermedades Metabólicas Asociadas (CIBERDEM), Instituto de Salud Carlos III, Málaga, Spain; 4 CNRS UMR8576 – UGSF – Unité de Glycobiologie Structurale et Fonctionnelle, Université de Lille, Lille, France; 5 Obesity and Metabolism Laboratory, Institute for Drug Research, School of Pharmacy, Faculty of Medicine, The Hebrew University of Jerusalem, Jerusalem, Israel; 6 Laboratory of Clinical Investigation, National Institute on Aging, National Institutes of Health, Baltimore, MD USA; 7 Departamento de Medicina y Cirugía, Facultad de Medicina, Universidad de Málaga, Málaga, Spain; 8 Grupo de Trabajo de Investigación Básica en Diabetes, Sociedad Española de Diabetes, Madrid, Spain

**Correction: Diabetologia (2024) 67:1877-1896**

10.1007/s00125-024-06193-6

Acknowledgment of funding for the project PID2021-128926OA-I00 by MICIU/AEI/10.13039/501100011033 and UE was omitted in the Funding section.

The original article has been corrected.

