# Supplementary material for: Pharmaceutical targeting of the cannabinoid type 1 receptor impacts the crosstalk between immune cells and islets to reduce insulitis in humans
Source: Diabetologia. 2024 Jun 12;67(9):1877–96. doi: 10.1007/s00125-024-06193-6 (PMC11410908; doi:10.1007/s00125-024-06193-6)
Supplement: Supplementary file 1 — ESM 1 (PDF 1746 KB) [file 125_2024_6193_MOESM1_ESM.pdf]

ESM Table 1. Oligonucleotides

<table>
<tr><th>Gene</th><th colspan="2">5’-3’ Forward Primer</th><th colspan="2">5’-3’ Reverse Primer</th></tr>
<tr><td>ABDH6</td><td colspan="2">TGTCCGCATCCCTCATAA</td><td colspan="2">GTCGGAACCTTGATCTTGTC</td></tr>
<tr><td>ABDH12</td><td colspan="2">CTTCCAGCTTGGCAGAAA</td><td colspan="2">CTGTAGCCAAGGTCTGAATG</td></tr>
<tr><td>ATF4</td><td colspan="2">TATGAGCCCAGAGTCCTATC</td><td colspan="2">CAGGAGGATCGTAAGGTTTG</td></tr>
<tr><td>ATF6</td><td colspan="2">AACCTGCACCCACTADAGGE</td><td colspan="2">CAAGGACTGGCTGAGCAG</td></tr>
<tr><td>CCL2</td><td colspan="2">CAGCAAGTGTCCCAAAGAAG</td><td colspan="2">GTCTTCGGAGTTTGGGTTTG</td></tr>
<tr><td>CD4</td><td colspan="2">GTACAGCTTCCCAGAAGAAG</td><td colspan="2">CAGCTTGGATGGACCTTTAG</td></tr>
<tr><td>CXCL10</td><td colspan="2">GCATTCAAGGAGTACCTCTCTC</td><td colspan="2">CAGACATCTCTTCTCACCCTTC</td></tr>
<tr><td>DAGLA</td><td colspan="2">TAACCTGCGGACCTACAA</td><td colspan="2">GCAGCAGAGGAACACTTT</td></tr>
<tr><td>DAGLB</td><td colspan="2">GCAGTTACTTGATCGTCCTC</td><td colspan="2">CGGTCCAGGGTTACAAATC</td></tr>
<tr><td>DDIT3</td><td colspan="2">CTTGGCTGACTGAGGAGGAG</td><td colspan="2">CTGGGGAATGACCACTCTGT</td></tr>
<tr><td>FAAH</td><td colspan="2">CAACTGTGTGACCTCCTATC</td><td colspan="2">ATGAACCGCAGACACAAC</td></tr>
<tr><td>GAPDH</td><td colspan="2">CATCCTGGGCTACACTGAGC</td><td colspan="2">AAAGTGGTCGTTGAGGGCAA</td></tr>
<tr><td>GPR78 (BiP)</td><td colspan="2">TTGACCAGCGTGTCATGGAA</td><td colspan="2">CGGAGTTTTGCACAGCTCT</td></tr>
<tr><td>HLA-ABC</td><td colspan="2">GAGAACGGGAAGGAGACGC</td><td colspan="2">CATCTCAGGGTGAGGGGCT</td></tr>
<tr><td>ICAM1</td><td colspan="2">GTTGTTGGGCATAGAGACC</td><td colspan="2">GCTCAGTTCATACACCTTCC</td></tr>
<tr><td>IFNG</td><td colspan="2">GAATGTCCAACGCAAAGC</td><td colspan="2">CCTCGAAACAGCATCTGAC</td></tr>
<tr><td>IL1B</td><td colspan="2">ATCTCCGACCACCACTAC</td><td colspan="2">AGGTGCTCAGGTCATTCT</td></tr>
<tr><td>MAGL</td><td colspan="2">AGGTGCCTACCATGTTCT</td><td colspan="2">TGGCTGTCCTTTGAGAGA</td></tr>
<tr><td>NAPEPLD</td><td colspan="2">TGTGGCTGTGAGAATGTG</td><td colspan="2">GTGCTGGGAAGGTGTAAAG</td></tr>
<tr><td>PDL1</td><td colspan="2">CACCAATTCCAAGAGAGAGG</td><td colspan="2">AGAGGTAGTTCTGGGATGAC</td></tr>
<tr><td>RPLP0</td><td colspan="2">CCTGAGTGATGTGCAGCTGA</td><td colspan="2">CCATTGTCGAACACCTGCTG</td></tr>
<tr><td>SLC2A1</td><td colspan="2">GTGCAGCAGECTGTGTATGC</td><td colspan="2">GGCCACGATGCTCAGATAGG</td></tr>
<tr><td>SLC2A2</td><td colspan="2">TGTGCTGGGTTCCTTCCAGT</td><td colspan="2">G999TTGGTTTTGGGTTCAT</td></tr>
<tr><td>TNF</td><td colspan="2">CTCTTCTCCTTCCTGATCGT</td><td colspan="2">CAGAGGGCTGATTAGAGAGA</td></tr>
<tr><td>CNR1</td><td colspan="4" rowspan="4">From [5]</td></tr>
<tr><td>CNR2</td></tr>
<tr><td>CNR1b</td></tr>
<tr><td>ACTB</td></tr>
<tr><th colspan="5">Endpoint PCR</th></tr>
<tr><td>CNB1e4</td><td colspan="2">CCAGCAGACCAGGTGAACAT</td><td colspan="2">GTCGATGGCTGTGAGGAACA</td></tr>
<tr><td></td><th>5’-3’ Forward Primer</th><th colspan="2">5’-3’ Reverse Primer</th><th>Probe</th></tr>
<tr><td>AAV2-ITR</td><td>GGAACCCCTAGTGATGGAGTT</td><td colspan="2">CGGCCTCAGTGAGCGA</td><td>FAM-CACTCCCTCTCTGCGCGCTCG-BHQ1</td></tr>
<tr><th colspan="5">ssDNA for CB1 gRNA cloning</th></tr>
<tr><td colspan="5">tatatcttgtggaaaggacgaaacaccgctggcggtggcagacctcctggttttagtactctggaaacagaatcta</td></tr>
</table>

ESM Table 2. MRM transitions for ECs measurements in ESI+ and ESI-.

Analyte	Molecular ion $[M+H]^+$	Fragment [m/z]	DP [volts]	CE [volts]	CXP [volts]
2-AG	379.2	287.1 (quantifier)	70	19	14
		91 (qualifier)	70	67	10
AEA	348.2	287.1 (quantifier)	26	13	16
		62 (qualifier)	26	13	8
d_4-AEA	352.3	287.1 (quantifier)	66	15	20
		66 (qualifier)	66	21	8

[a] 2-AG = 2-arachidonoylglycerol; AEA = anandamide; DP = declustering potential; CE = collision energy; CXP = collision cell exit potential

ESM Fig. 1

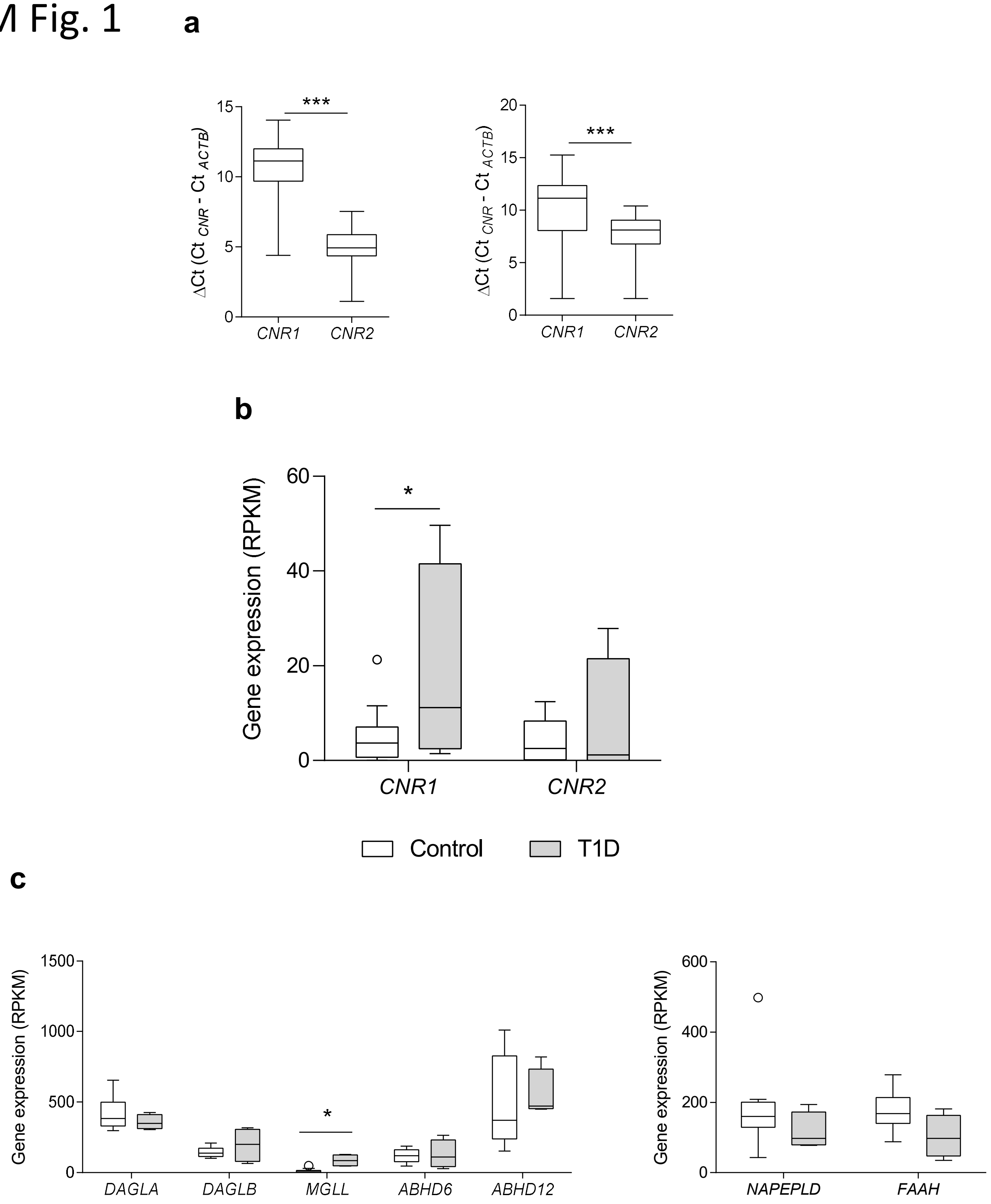

ESM Figure 1. Sorted islet beta cells from T1D donors have higher *CNR1* expression levels compared to those of healthy donors. (**a**) Comparison of *CNR1* and *CNR2* expression levels in PBMCs (left graph) and $CD4^+$ T cells (right graph) shown as the ΔCt of the specific gene and *ACTB* (higher ΔCt equals to lower expression). Gene expression of (**b**) *CNR1* and *CNR2* mRNA and (**c**) genes involved in 2-AG and AEA metabolism in sorted beta cells from islets of healthy donors (control; n = 10) and donors with type 1 diabetes (T1D; n = 4). The expression is shown as RPKM in Box & Whiskers Tukey graph. Data from GSE121863. Significance by 2-way ANOVA – Sidak post hoc test; * $p < 0.05$.

ESM Fig. 2

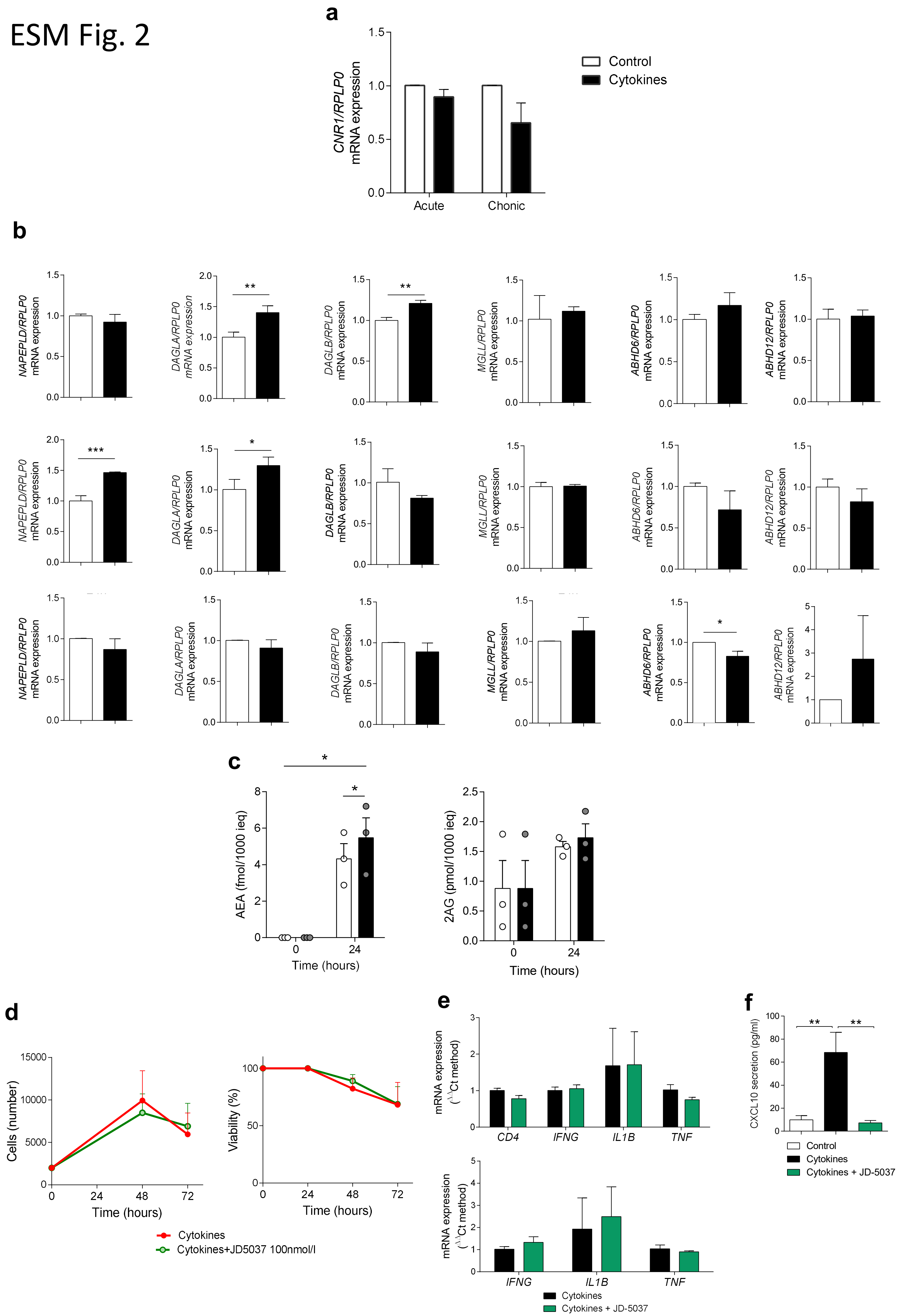

a
CNR1/RPLP0 mRNA expression
Acute
Chonic
Control
Cytokines
b
NAPEPLD/RPLP0 mRNA expression
DAGLA/RPLP0 mRNA expression
DAGLB/RPLP0 mRNA expression
MGLL/RPLP0 mRNA expression
ABHD6/RPLP0 mRNA expression
ABHD12/RPLP0 mRNA expression
c
AEA (fmol/1000 ieq)
2AG (pmol/1000 ieq)
Time (hours)
d
Cells (number)
Viability (%)
Time (hours)
Cytokines
Cytokines+JD5037 100nmol/l
e
mRNA expression
CD4
IFNG
IL1B
TNF
Cytokines
Cytokines + JD-5037
f
CXCL10 secretion (pg/ml)
Control
Cytokines
Cytokines + JD-5037

ESM Figure 2. Human islets in culture secrete high levels of endocannabinoids independently of cytokines. Expression of (**a**) *CNR1* mRNA in islets cultured for 2 (acute) or 24 h (chronic) with or without cytokines. The expression of *RPLP0* was used as a control. Data are mean ± SEM, $n = 4$ donors. Significance by 2-way ANOVA – Sidak post hoc test. (**b**) Expression of endocannabinoid enzymes after 1 (top graphs), 4 (middle graphs) or 24 hours (bottom graphs) of exposure to cytokines. Levels of (**c**) 2-arachidonoyl glycerol (2-AG) and anandamide (AEA) secreted to the media from islets (1000 IEQ) before and after 24 and 48 h of culture with or without cytokines. Data are mean ± SEM, $n = 3$ donors. Paired Significance by 2-way ANOVA – Sidak post hoc test; * $p < 0.05$, ** $p < 0.01$. PBMCs were treated with cytokines in the presence of vehicle or JD-5037 (100 nmol/l) and (**d**) proliferation (left) and viability (right) of PBMCs, as well as (**e**) mRNA expression levels for proinflammatory cytokines, were determined. The expression of *GAPDH* (top graph) or *CD4* (bottom graph) was used as a control. (**f**) Secretion of CXCL10 by PBMCs into the media. Data are mean ± SEM, $n = 3$ donors. Significance by 1-way ANOVA – Tukey post hoc.

ESM Fig. 3

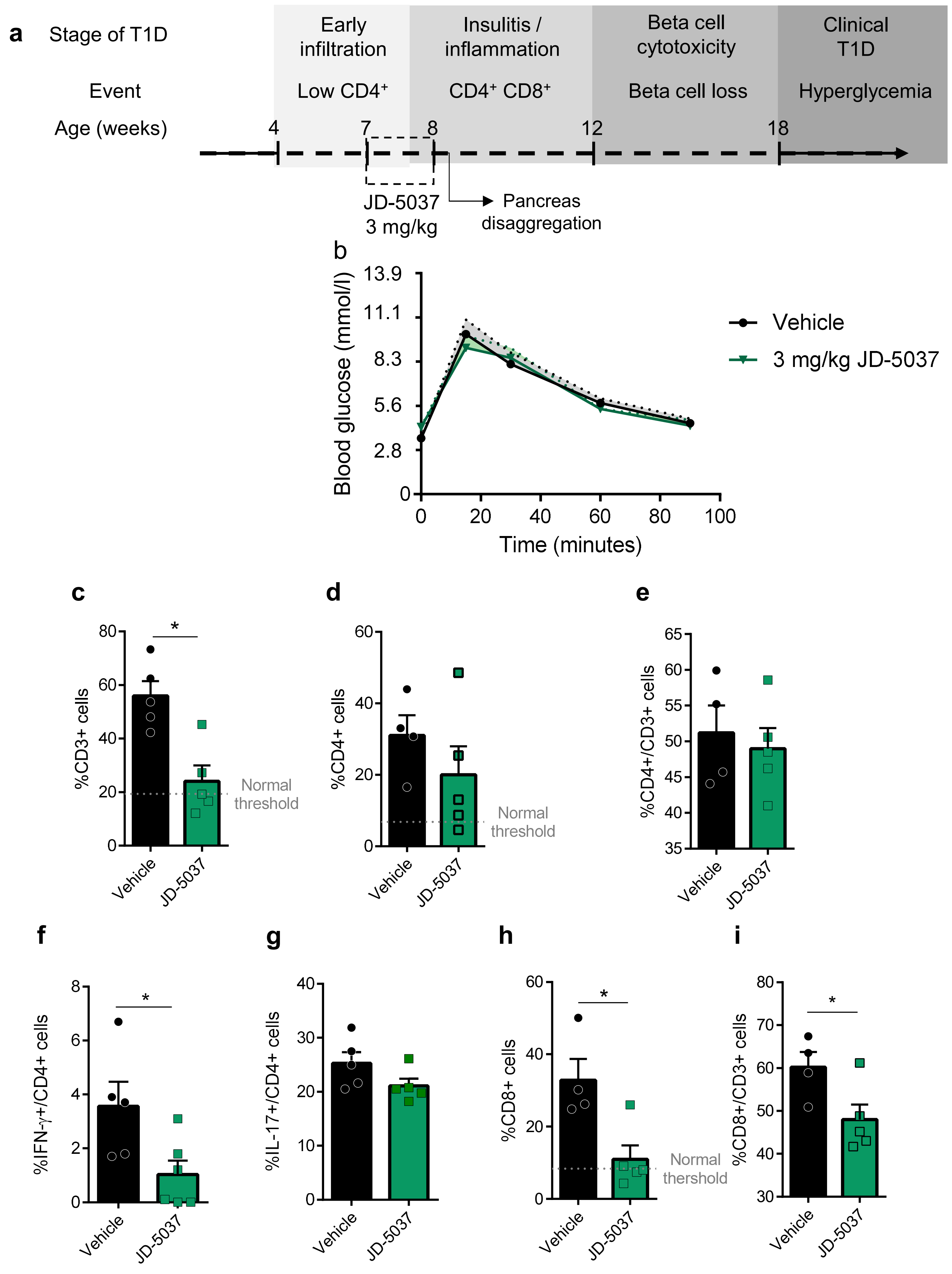

a
Stage of T1D
Early infiltration
Insulitis / inflammation
Beta cell cytotoxicity
Clinical T1D
Event
Low CD4⁺
CD4⁺ CD8⁺
Beta cell loss
Hyperglycemia
Age (weeks)
4
7
8
12
18
JD-5037
3 mg/kg
Pancreas disaggregation
b
Blood glucose (mmol/l)
13.9
11.1
8.3
5.6
2.8
0
0
20
40
60
80
100
Time (minutes)
Vehicle
3 mg/kg JD-5037
c
%CD3+ cells
*
Normal threshold
Vehicle
JD-5037
d
%CD4+ cells
Normal threshold
e
%CD4+/CD3+ cells
f
%IFN-γ+/CD4+ cells
g
%IL-17+/CD4+ cells
h
%CD8+ cells
Normal thershold
i
%CD8+/CD3+ cells

ESM Fig. 3

j

Vehicle-treated NOD JD5037-treated NOD

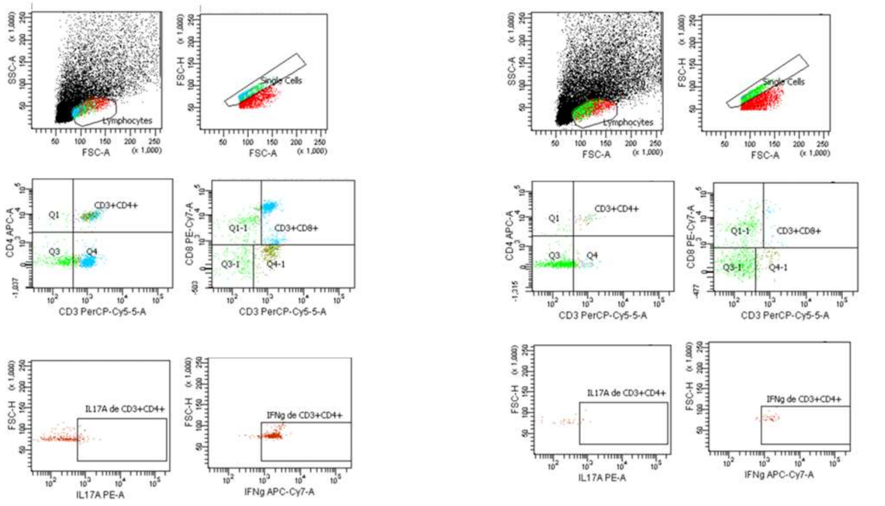

ESM Figure 3. Selective peripheral CB1R blockade arrests initiation of insulitis in NOD mice. (**a**) Schema of the experimental procedure. (**b**) Intraperitoneal glucose tolerance test after 1-week treatment with vehicle or JD-5037. Quantification by flow cytometry of various immune cell subpopulations (**c-j**) in infiltrated lymphocytes in the pancreas of NOD mice treated with vehicle or JD-5037. Data are mean ± SEM and individual values. The dotted line represents the values obtained in 4-week-old NOD mice. Significance by Student's t-test; * $p < 0.05$, $n = 5$-6 mice/group.

ESM Fig. 4

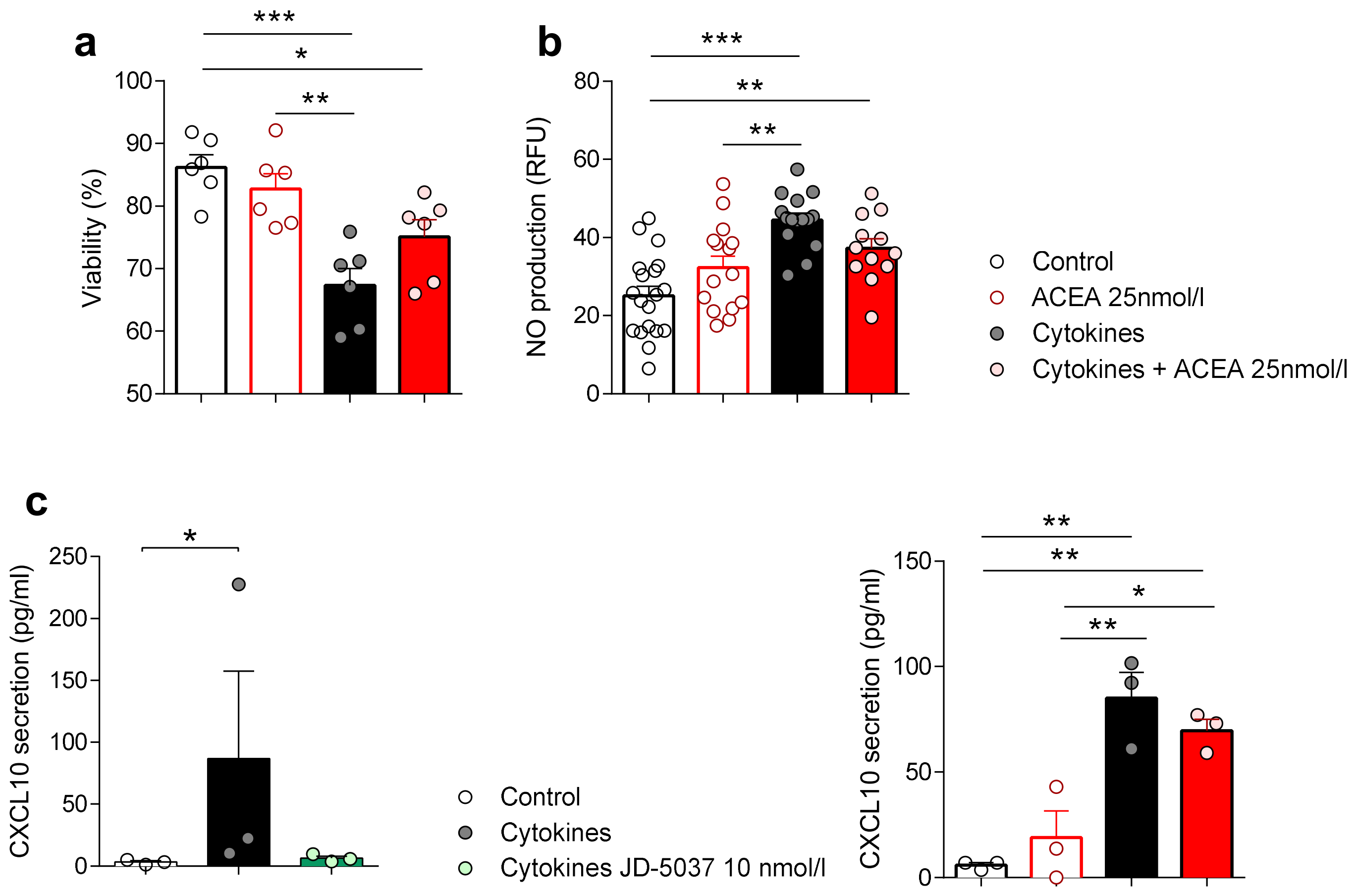

ESM Figure 4. ACEA and cytokines do not have a synergistic effect on islets. (**a**) Islet viability and (**b**) NO production in islets treated with vehicle of ACEA (25 nmol/l) and with a mix of cytokines. (**c**) Islet CXCL10 secretion into the media. Significance by 1-way ANOVA – Tukey post hoc test; * $p < 0.05$, ** $p < 0.01$, *** $p < 0{,}001$, n = 2-3 donors.

ESM Fig. 5

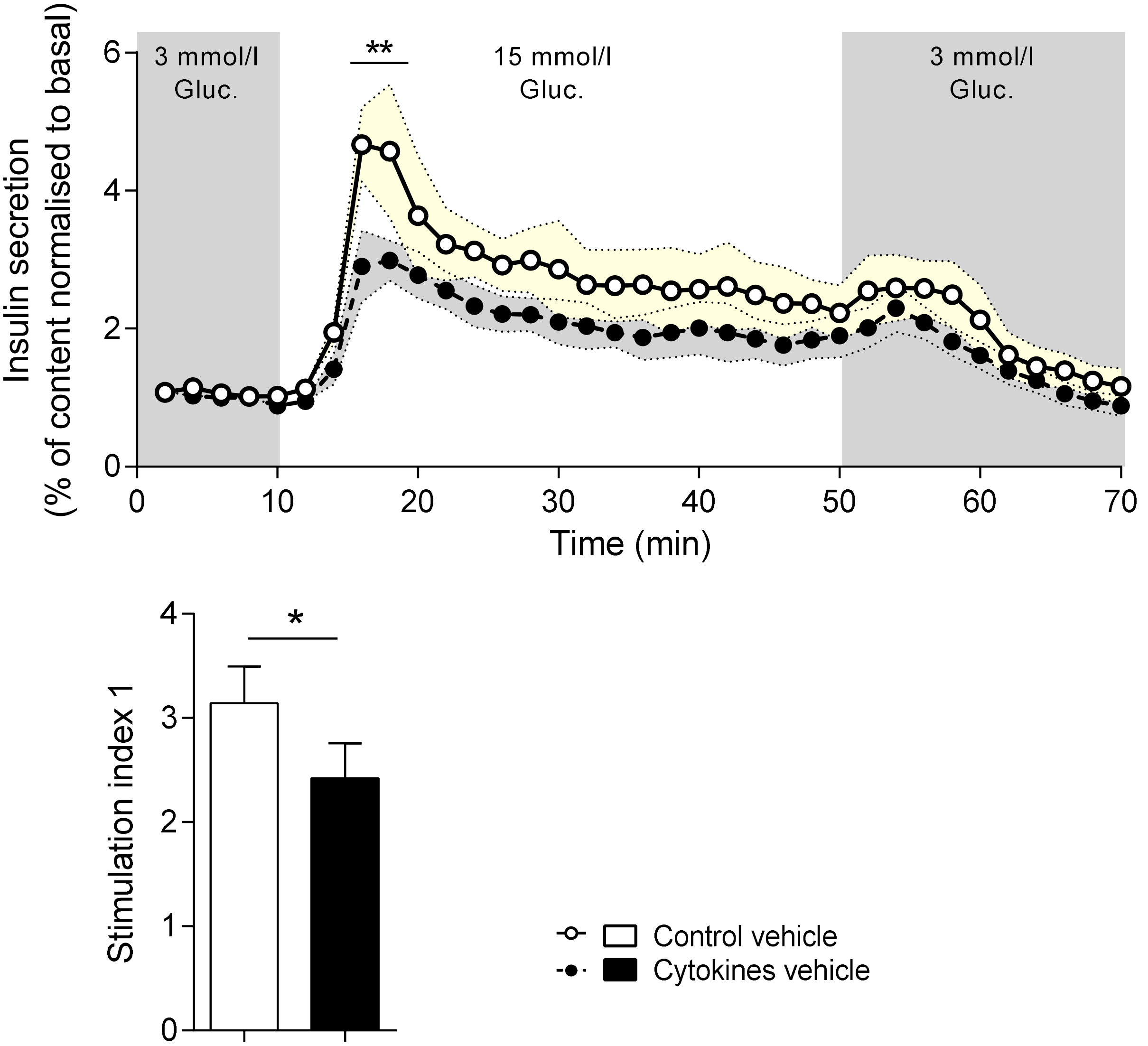

ESM Figure 5. Cytokines induce beta cell dysfunction. Dynamic GSIS after 24 h of insult with a mix of cytokines, and stimulation index, *n* = 6 donors. Data are mean ± SEM (shadowed or error bars). * $p < 0.05$, ** $p < 0.01$ between cytokine and control groups.

Checklist for reporting human islet preparations used in research

Adapted from Hart NJ, Powers AC (2018) Progress, challenges, and suggestions for using human islets to understand islet biology and human diabetes. Diabetologia https://doi.org/10.1007/s00125-018-4772-2

Islet preparation	1	2	3	4	5	6	7	8[a]
**MANDATORY INFORMATION**								
Unique identifier	IG14	IG15	IG16	IG18	IG19	IG20	IG21	IG22
Donor age (years)	39	58	66	68	45	59	61	46
Donor sex (M/F)	F	M	M	F	M	F	M	M
Donor BMI (kg/m^2)	36	26.7	25.6	28.9	26.8	29.2	31.3	28.1
Donor HbA_{1c} (%)	5.2	5.8	5.4	5.6	5.4	5.8	7.8	5.8
Donor HbA_{1c} (mmol/mol)	33	40	36	38	36	40	62	40
Origin/source of islets[b]	Inserm UMR1190	Inserm UMR1190	Inserm UMR1190	Inserm UMR1190	Inserm UMR1190	Inserm UMR1190	Inserm UMR1190	Inserm UMR1190
Islet isolation centre	CHU, Univ. Lille	CHU, Univ. Lille	CHU, Univ. Lille	CHU, Univ. Lille	CHU, Univ. Lille	CHU, Univ. Lille	CHU, Univ. Lille	CHU, Univ. Lille
Donor history of diabetes? Please select yes/no from drop down list	No	No	No	No	No	No	Yes	No
**If Yes, complete the next two lines if this information is available**								
Diabetes duration (years)								
Glucose-lowering therapy at time of death[c]							No	
**RECOMMENDED INFORMATION**								
Donor cause of death								
Warm ischaemia time (h)								

Cold ischaemia time (h)								
Estimated purity (%)	70	75	80	80	90	80	80	80
Estimated viability (%)	95	91.4	92.9	92.3	94	93.9	91.3	88.5
Total culture time (h)[d]	24H-Multiple timepoints	36H-Multiple timepoints	36H-Multiple timepoints	24H-Multiple timepoints	18H-Multiple timepoints	24H-Multiple timepoints	24H-Multiple timepoints	24H-Multiple timepoints
Glucose-stimulated insulin secretion or other functional measurement[e]	S1/B 3.61	S1/B 2.29	S1/B 2.43	S1/B 2.25	S1/B 1.83	S1/B 3.93	S1/B 2.01	S1/B 5.14
Handpicked to purity? Please select yes/no from drop down list	No	No	No	No	No	No	No	No
Additional notes	CRISPR/Cas9	CRISPR/Cas9	Response to cytokines	Response to cytokines	Response to cytokines	Response to cytokines	CRISPR/Cas9	CRISPR/Cas9

[a]If you have used more than eight islet preparations, please complete additional forms as necessary
[b]For example, IIDP, ECIT, Alberta IsletCore
[c]Please specify the therapy/therapies
[d]Time of islet culture at the isolation centre, during shipment and at the receiving laboratory
[e]Please specify the test and the results

Islet preparation	1	2	3	4	5	6	7	8[a]
**MANDATORY INFORMATION**								
Unique identifier	EW1	IG24	IG25	IG26	IG27	IG28	IG30	
Donor age (years)	28	49	26	70	29	56	50	
Donor sex (M/F)	M	M	F	M	M	M	F	
Donor BMI (kg/m^2)	28.1	20.9	36.7	26.6	43.3	24	30	
Donor HbA_{1c} (%)	5.7	5.5	5.6	5.6	5.8	5.0	5.4	
Donor HbA_{1c} (mmol/mol)	39	37	38	38	40	31	36	
Origin/source of islets[b]	Inserm UMR1190	Inserm UMR1190	Inserm UMR1190	Inserm UMR1190	Inserm UMR1190	Inserm UMR1190	Inserm UMR1190	
Islet isolation centre	CHU, Univ. Lille	CHU, Univ. Lille	CHU, Univ. Lille	CHU, Univ. Lille	CHU, Univ. Lille	CHU, Univ. Lille	CHU, Univ. Lille	
Donor history of diabetes? Please select yes/no from drop down list	No	No	No	No	No	No	No	
**If Yes, complete the next two lines if this information is available**								
Diabetes duration (years)								
Glucose-lowering therapy at time of death[c]								
**RECOMMENDED INFORMATION**								
Donor cause of death								
Warm ischaemia time (h)								
Cold ischaemia time (h)								

Estimated purity (%)	90	90	70	70	50	40	70	
Estimated viability (%)	93.1	96.7	72	96	91.8	91.9	93	
Total culture time (h)[d]	24H-Multiple timepoints	72H-Multiple timepoints	36H-Multiple timepoints	20H-Multiple timepoints	24H-Multiple timepoints	24H-Multiple timepoints	24H-Multiple timepoints	
Glucose-stimulated insulin secretion or other functional measurement[e]	S1/B 1.05	S1/B 5.88	S1/B 5.20	S1/B 3.15	S1/B 2.74	S1/B 0.96	S1/B 3.43	
Handpicked to purity? Please select yes/no from drop down list	No	No	No	No	Yes	Yes	No	
Additional notes	Response to cytokines	CRISPR/Cas9	CRISPR/Cas9	CRISPR/Cas9	CRISPR/Cas9	CRISPR/Cas9	CRISPR/Cas9	

[a]If you have used more than eight islet preparations, please complete additional forms as necessary
[b]For example, IIDP, ECIT, Alberta IsletCore
[c]Please specify the therapy/therapies
[d]Time of islet culture at the isolation centre, during shipment and at the receiving laboratory
[e]Please specify the test and the results

Islet preparation	1	2	3	4	5	6	7	8[a]
**MANDATORY INFORMATION**								
Unique identifier	IG3	IG4	IG6	IG8	IG9	IG10	IG11	IG12
Donor age (years)	55	33	56	46	44	50	61	73
Donor sex (M/F)	F	M	M	M	M	M	F	M
Donor BMI (kg/m^2)	27.5	24.5	25.2	21.1	27.8	24.7	23.3	26
Donor HbA_{1c} (%)	5.5	5.1	5.3		5.5	5.4	5.6	5.7
Donor HbA_{1c} (mmol/mol)	37	32	34		37	36	38	39
Origin/source of islets[b]	Inserm UMR1190	Inserm UMR1190	Inserm UMR1190	Inserm UMR1190	Univ. Maastricht	Inserm UMR1190	Inserm UMR1190	Inserm UMR1190
Islet isolation centre	CHU, Univ. Lille	CHU, Univ. Lille	CHU, Univ. Lille	CHU, Univ. Lille		CHU, Univ. Lille	CHU, Univ. Lille	CHU, Univ. Lille
Donor history of diabetes? Please select yes/no from drop down list	No	No	No	Yes	No	No	No	No
**If Yes, complete the next two lines if this information is available**								
Diabetes duration (years)								
Glucose-lowering therapy at time of death[c]								
**RECOMMENDED INFORMATION**								
Donor cause of death								
Warm ischaemia time (h)								
Cold ischaemia time (h)								
Estimated purity (%)	80	90	80	90	90	80	80	90

Estimated viability (%)	96	97	98.7	97	97.7	95	98	97
Total culture time (h)[d]	24H-Multiple timepoints	96H-Multiple timepoints	24H-Multiple timepoints	24H-Multiple timepoints	48H-Multiple timepoints	36H-Multiple timepoints	48H-Multiple timepoints	24H-Multiple timepoints
Glucose-stimulated insulin secretion or other functional measurement[e]	S1/B 3.69	S1/B 1.62	S1/B 6.76	S1/B 4.7	S1/B 1.97	S1/B 13.67	S1/B 3.68	S1/B 1.24
Handpicked to purity? Please select yes/no from drop down list	No	No	No	No	No	No	No	No
Additional notes	Response to cytokines	Response to cytokines	Response to cytokines	Response to cytokines	Response to cytokines	Response to cytokines	Response to cytokines	Response to cytokines

[a]If you have used more than eight islet preparations, please complete additional forms as necessary
[b]For example, IIDP, ECIT, Alberta IsletCore
[c]Please specify the therapy/therapies
[d]Time of islet culture at the isolation centre, during shipment and at the receiving laboratory
[e]Please specify the test and the results

Islet preparation	1	2	3	4	5	6	7	8[a]
**MANDATORY INFORMATION**								
Unique identifier	IG33	IG34						
Donor age (years)	58	65						
Donor sex (M/F)	F	F						
Donor BMI (kg/m^2)	24	51						
Donor HbA_{1c} (%)	6.1	5						
Donor HbA_{1c} (mmol/mol)	43	31						
Origin/source of islets[b]	Inserm UMR1190	Inserm UMR1190						
Islet isolation centre	CHU, Univ. Lille	CHU, Univ. Lille						
Donor history of diabetes? Please select yes/no from drop down list	No	No						
**If Yes, complete the next two lines if this information is available**								
Diabetes duration (years)								
Glucose-lowering therapy at time of death[c]								
**RECOMMENDED INFORMATION**								
Donor cause of death								
Warm ischaemia time (h)								
Cold ischaemia time (h)								
Estimated purity (%)	70							

Estimated viability (%)	92							
Total culture time (h)[d]	24H-24H	24H-24H						
Glucose-stimulated insulin secretion or other functional measurement[e]								
Handpicked to purity? Please select yes/no from drop down list	No	No						
Additional notes	Response to ACEA	Response to ACEA						

[a]If you have used more than eight islet preparations, please complete additional forms as necessary
[b]For example, IIDP, ECIT, Alberta IsletCore
[c]Please specify the therapy/therapies
[d]Time of islet culture at the isolation centre, during shipment and at the receiving laboratory
[e]Please specify the test and the results
